# Supplementary material for: Learning the intrinsic dynamics of spatio-temporal processes through Latent Dynamics Networks
Source: Nat Commun. 2024 Feb 28;15:1834. doi: 10.1038/s41467-024-45323-x (PMC11258335; doi:10.1038/s41467-024-45323-x)
Supplement: Supplementary file 1 — Supplementary Information [file 41467_2024_45323_MOESM1_ESM.pdf]

Supplementary Information

Learning the intrinsic dynamics of spatio-temporal processes through Latent Dynamics Networks

Francesco Regazzoni[1,*], Stefano Pagani[1], Matteo Salvador[1,2], Luca Dede'[1], Alfio Quarteroni[1,3]

[1] MOX, Department of Mathematics, Politecnico di Milano, Milan, Italy
[2] Institute for Computational and Mathematical Engineering, Stanford University, California, USA
[3] École Polytechnique Fédérale de Lausanne, Lausanne, Switzerland (*Professor Emeritus*)

[*] *Corresponding author* (francesco.regazzoni@polimi.it)

1 Error metrics

To evaluate the generalization accuracy, we test trained models on unseen data, that is on samples belonging to a test set denoted by $\mathcal{S}_{\text{test}}$. Specifically, we employ two metrics.

Normalized root mean square error (NRMSE) is obtained as the square root of the mean of the squares of the normalized errors obtained on the test set:

$$\text{NRMSE} = \sqrt{\sum\!\!\!\!\!\!\!\!\;\int_{i\in\mathcal{S}_{\text{test}}} \sum\!\!\!\!\!\!\!\!\;\int_{\tau\in\mathscr{T}^i} \sum\!\!\!\!\!\!\!\!\;\int_{\boldsymbol{\xi}\in\mathscr{P}^i_\tau} \frac{\|\widetilde{\mathbf{y}}_i(\boldsymbol{\xi},\tau)-\mathbf{y}_i(\boldsymbol{\xi},\tau)\|^2}{y^2_{\text{norm}}}}.$$

Pearson dissimilarity $(1-\rho)$ is defined from the Pearson correlation coefficient ρ:

$$\rho = \frac{\displaystyle\sum_{i\in\mathcal{S}_{\text{test}}} \sum_{\tau\in\mathscr{T}^i} \sum_{\boldsymbol{\xi}\in\mathscr{P}^i_\tau} (\widetilde{\mathbf{y}}_i(\boldsymbol{\xi},\tau)-\overline{\widetilde{\mathbf{y}}})\cdot(\mathbf{y}_i(\boldsymbol{\xi},\tau)-\overline{\mathbf{y}})}{\sqrt{\displaystyle\sum_{i\in\mathcal{S}_{\text{test}}} \sum_{\tau\in\mathscr{T}^i} \sum_{\boldsymbol{\xi}\in\mathscr{P}^i_\tau} \|\widetilde{\mathbf{y}}_i(\boldsymbol{\xi},\tau)-\overline{\widetilde{\mathbf{y}}}\|^2 \sum_{i\in\mathcal{S}_{\text{test}}} \sum_{\tau\in\mathscr{T}^i} \sum_{\boldsymbol{\xi}\in\mathscr{P}^i_\tau} \|\mathbf{y}_i(\boldsymbol{\xi},\tau)-\overline{\mathbf{y}}\|^2}}$$

where we denote the average outputs as

$$\overline{\mathbf{y}} = \sum\!\!\!\!\!\!\!\!\;\int_{i\in\mathcal{S}_{\text{test}}} \sum\!\!\!\!\!\!\!\!\;\int_{\tau\in\mathscr{T}^i} \sum\!\!\!\!\!\!\!\!\;\int_{\boldsymbol{\xi}\in\mathscr{P}^i_\tau} \mathbf{y}_i(\boldsymbol{\xi},\tau)$$

$$\overline{\widetilde{\mathbf{y}}} = \sum\!\!\!\!\!\!\!\!\;\int_{i\in\mathcal{S}_{\text{test}}} \sum\!\!\!\!\!\!\!\!\;\int_{\tau\in\mathscr{T}^i} \sum\!\!\!\!\!\!\!\!\;\int_{\boldsymbol{\xi}\in\mathscr{P}^i_\tau} \widetilde{\mathbf{y}}_i(\boldsymbol{\xi},\tau)$$

We remark that both metrics (RMSE and $1-\rho$) are robust with respect to multiplicative rescaling of the outputs. For both of them, the smaller the value of the metric, the higher the accuracy of the predictions.

2 Alternative methods

In this section, we describe alternative methods to LDNets, whose performance is compared in the main text and in Sec. 3. Some of these methods are based on a space discretization of the solution field.

We consider hence a space-discretization operator $\mathcal{A}\colon \mathcal{Y}\to\mathbb{R}^{N_h}$, with $N_h \gg 1$. The operator $\mathcal{A}$ typically consists of a point-wise evaluation of $\mathbf{y}$ on a grid of points (e.g. the nodes of a computational mesh), or of an expansion with respect to a Finite Element basis or to a Fourier basis. The subscript h refers to the characteristic size associated with the discretization (e.g., the mesh element size for a mesh-based discretization; the sampling period for a discretization based on the discrete Fourier transform). Hence, we

write $\mathbf{Y}(t) = \mathcal{A}(\mathbf{y}(\cdot, t)) \in \mathbb{R}^{N_h}$. The space-discretization operator is typically accompanied by a discrete-to-continuous operator $\mathcal{A}' \colon \mathbb{R}^{N_h} \to \mathcal{Y}$, such that $\mathbf{y}(\cdot, t) \simeq \mathcal{A}'(\mathbf{Y}(t))$.

Let us consider, for simplicity, the case when the discretization operator is associated with the evaluation of the output field on a grid of points $\boldsymbol{\xi}_1, \boldsymbol{\xi}_2, \dots, \boldsymbol{\xi}_{N_{\text{nodes}}}$. Then we have

$$\mathbf{Y}_i(\tau) = (\mathbf{y}_i(\boldsymbol{\xi}_1, \tau), \mathbf{y}_i(\boldsymbol{\xi}_1, \tau), \dots, \mathbf{y}_i(\boldsymbol{\xi}_{N_{\text{nodes}}}, \tau)) \quad \text{for } i \in \mathcal{S}_{\text{train}}, \tau \in \mathscr{T}^i$$

In this case, we have $N_h = d_{\mathbf{y}} N_{\text{nodes}}$. We are thus restricting ourselves to the case when the training dataset is such that $\mathscr{P}_\tau^i \equiv \mathscr{P} := (\boldsymbol{\xi}_1, \boldsymbol{\xi}_2, \dots, \boldsymbol{\xi}_{N_{\text{nodes}}})$. If, instead, observation points vary between samples or between time steps, interpolation would be required.

2.1 Projection-based reduced-order models

Projection-based reduced-order models (ROMs) allow the efficient simulation of complex spatio-temporal systems for many different queries of the input $\mathbf{u}(t)$ [2–5, 12, 17]. They leverage Galerkin or Petrov-Galerkin projection of the PDE system onto problem-specific linear manifolds to reduce the complexity of high-fidelity discretization, deliberately rich in degrees of freedom for the sake of accurately approximating the PDE solution. Here, to ease the notation, we only consider the case in which the model output coincides with the FOM state ($\mathbf{y} \equiv \mathbf{z}$), that is the observation operator is the identity function. The case when the operator differs from the identity is a straightforward generalization of the one considered here.

Projection-based ROMs are intrusive, as they require manipulation of the discretization of FOM. Their construction first involves dimensionality reduction techniques applied to pre-computed samples of the discretized FOM state:

$$\mathbf{Z}(t) = \mathcal{A}(\mathbf{z}(\cdot, t)) \in \mathbb{R}^{N_h},$$

collected by numerically approximating the space-discrete counterpart of the FOM, for different samples of the input $\mathbf{u}(t)$ in the training set:

$$\begin{cases} \dfrac{d}{dt}\mathbf{Z}(t) = \mathbf{F}(\mathbf{Z}(t), \mathbf{u}(t)) & \text{for } t \in (0, T] \\ \mathbf{Z}(t) = \mathbf{Z}_0 := \mathcal{A}(\mathbf{z}_0), \end{cases} \tag{1}$$

where $\mathbf{F}$ is a suitable discretization of the differential operator $\mathcal{F}$. Solution snapshots are stored into a matrix

$$Z = \{\mathcal{A}(\mathbf{z}_i(\cdot, \tau))\}_{i \in \mathcal{S}_{\text{train}}}^{\tau \in \mathscr{T}^i} \in \mathbb{R}^{N_h \times N_{\text{snapshots}}}$$

from which $d_{\mathbf{s}} \ll N_h$ basis functions $\boldsymbol{\phi}_k$, $k = 1, \dots, d_{\mathbf{s}}$ are extracted by proper orthogonal decomposition (POD), or other linear dimensionality reduction techniques, like reduced-basis greedy algorithm [12, 18]. This allows expressing the discretized state compactly as

$$\mathbf{Z}(t) \approx V\mathbf{s}(t) = \sum_{k=1}^{d_{\mathbf{s}}} s_k(t) \boldsymbol{\phi}_k, \tag{2}$$

where $V = [\boldsymbol{\phi}_1 | \dots | \boldsymbol{\phi}_{d_{\mathbf{s}}}]$ is the transformation matrix, collecting the basis functions in its columns, and $\mathbf{s}(t) = [s_1(t), \dots, s_{d_{\mathbf{s}}}(t)]^T$ is the reduced state, a vector made of the coefficients s_k associated with each basis function. In this context, the solution manifold associated with the latent space is thus the column space of the matrix V.

Given a test manifold spanned by $d_{\mathbf{s}}$ basis functions $\{\boldsymbol{\psi}_k\}_{k=1}^{d_{\mathbf{s}}}$ (collected into a matrix $W = [\boldsymbol{\psi}_1 | \dots | \boldsymbol{\psi}_{d_{\mathbf{s}}}]$), the projection of the dynamical system (1) generates a system of $d_{\mathbf{s}}$ equations for the reduced state of the form:

$$\begin{cases} \dfrac{d}{dt}\mathbf{s}(t) = W^T \mathbf{F}(V\mathbf{s}(t), \mathbf{u}(t)) & \text{for } t \in (0, T] \\ \mathbf{s}(0) = \mathbf{s}_0 := V^T \mathbf{Z}_0, \end{cases} \tag{3}$$

In this formulation, computational efficiency hinges on the form of the discretized operator $\mathbf{F}$. In the linear and affine cases, the dependence on the solution can be separated from the operator, generating a set or

pre-computable terms that ensure computational efficiency in the resolution. In the case nonlinearities are present in the model, instead, $\mathbf{F}$ requires the reduced state to be projected back onto the state space for its evaluation, before being projected onto the low-dimensional dynamics of (3). This double projection makes the computational costs of (3) comparable to the ones of (1). Hyper-reduction techniques based on interpolation and linear dimensionality reduction techniques provide a computationally efficient alternative for evaluating nonlinearities [7, 12, 18]. In our test case, we first separate the nonlinear part from the linear one:

$$\mathbf{F}(\mathbf{s}(t), \mathbf{u}(t)) = \mathbf{F}_{nl}(\mathbf{s}(t), \mathbf{u}(t)) + \mathbf{F}_{l}(\mathbf{s}(t), \mathbf{u}(t)),$$

and we then rely on the discrete version of the empirical interpolation method (DEIM), which employs an interpolation-based projection of the nonlinearity onto the span of sparsely sampled basis functions, precomputed with POD [7]. Specifically, the non-linear term $\mathbf{F}_{nl}$ is approximated by

$$\mathbf{F}_{nl}(\mathbf{s}(t), \mathbf{u}(t)) \approx U(P^T U)^{-1} P^T \mathbf{F}_{nl}(\mathbf{s}(t), \mathbf{u}(t)),$$

being U the transformation matrix, collecting POD basis functions of the nonlinear term, and P a sparse matrix that samples $\mathbf{F}_{nl}$ on a subset of interpolation indices. In the following, we will denote this model-order reduction method as POD-DEIM method.

The success and greatest limitation of projection-based ROM lies in being intrusively linked to high-fidelity discretization via a linear subspace. This, on the one hand, ensures the ROM consistency, with a reduced state directly mappable to the system state thanks to (2), and convergence to the FOM as the dimensionality of the reduced manifold increases. On the other hand, if the solution of the parametric problem entails large variability, as in the case of advection-dominated problems, the accuracy-efficiency balance is compromised by the curse of dimensionality.

2.2 Methods based on auto-encoders

For the reasons above, many researchers have shifted to the development of reduced-order models based on non-linear dimensionality reduction techniques, such as NN-based auto-encoders [6, 10, 14–16, 20]. In this framework, to reduce the dimensionality of the discretized output field $\mathbf{Y}$, we train an auto-encoder, with latent code dimension $d_{\mathbf{s}}$, by considering the following minimization problem:

$$\begin{aligned}(\mathbf{w}_{\text{enc}}^*, \mathbf{w}_{\text{dec}}^*) = \underset{\mathbf{w}_{\text{enc}}, \mathbf{w}_{\text{dec}}}{\operatorname{argmin}} \sum_{i \in \mathcal{S}_{\text{train}}} \sum_{\tau \in \mathscr{T}^i} \| \mathcal{NN}_{\text{dec}}(\mathcal{NN}_{\text{enc}}(\mathbf{Y}_i(\tau); \mathbf{w}_{\text{enc}}); \mathbf{w}_{\text{dec}}) - \mathbf{Y}_i(\tau) \|_{\mathbf{Y}}^2 \\ + \alpha_{\text{enc}} \mathcal{R}(\mathbf{w}_{\text{enc}}) + \alpha_{\text{dec}} \mathcal{R}(\mathbf{w}_{\text{dec}})\end{aligned}$$

where $\mathcal{NN}_{\text{enc}}$ and $\mathcal{NN}_{\text{dec}}$ (with trainable parameters $\mathbf{w}_{\text{enc}}$ and $\mathbf{w}_{\text{dec}}$, respectively) are the encoder and the decoder, respectively. For normalization purposes, to evaluate distances in the discrete space we employ the rescaled euclidean norm $\| \cdot \|_{\mathbf{Y}}^2 := \| \cdot \|^2 / (N_{\text{nodes}}\, y_{\text{norm}}^2)$.

Once the auto-encoder is trained, i.e. the parameters $\mathbf{w}_{\text{enc}}^*$ and $\mathbf{w}_{\text{dec}}^*$ are available, we compute the latent codes associated with each training sample $i \in \mathcal{S}_{\text{train}}$ and each observation time $\tau \in \mathscr{T}^i$, that we denote as

$$\hat{\mathbf{s}}_i(\tau) = \mathcal{NN}_{\text{enc}}(\mathbf{Y}_i(\tau); \mathbf{w}_{\text{enc}}^*) \in \mathbb{R}^{d_{\mathbf{s}}}.$$

At this stage, we train a second model (denoted by $\mathcal{RNN}_{\text{dyn}}$, with trainable parameters $\mathbf{w}_{\text{dyn}}$) to predict the dynamics of the latent codes $\hat{\mathbf{s}}_i(t)$ as a function of the inputs $\{\mathbf{u}_i(\tau)\}_{\tau \in \mathscr{S}^i}$:

$$\mathbf{w}_{\text{dyn}}^* = \underset{\mathbf{w}_{\text{dyn}}}{\operatorname{argmin}} \sum_{i \in \mathcal{S}_{\text{train}}} \sum_{t \in \mathscr{T}^i} \| \mathcal{RNN}_{\text{dyn}}(\{\mathbf{u}_i(\tau)\}_{\tau \in \mathscr{S}^i}, t; \mathbf{w}_{\text{dyn}}) - \hat{\mathbf{s}}_i(t) \|^2 + \alpha_{\text{dyn}} \mathcal{R}(\mathbf{w}_{\text{dyn}})$$

In this work, we consider two different architectures for $\mathcal{RNN}_{\text{dyn}}$, namely an ODE-Net [8] and an LSTM [13]. In both cases, we employ normalization layers as described in the main text.

Once both the auto-encoder $\mathcal{NN}_{\text{dec}} \circ \mathcal{NN}_{\text{enc}}$ and the dynamics network $\mathcal{RNN}_{\text{dyn}}$ have been trained, the predictions on the test set are obtained as follows, for $i \in \mathcal{S}_{\text{test}}$ and $t \in \mathscr{T}^i$:

$$\widetilde{\mathbf{Y}}_i(t) = \mathcal{NN}_{\text{dec}}(\mathcal{RNN}_{\text{dyn}}(\{\mathbf{u}_i(\tau)\}_{\tau \in \mathscr{S}^i}, t; \mathbf{w}_{\text{dyn}}^*); \mathbf{w}_{\text{dec}}^*),$$

and

$$\widetilde{\mathbf{y}}_i(\cdot,t) = \mathcal{A}'(\mathcal{NN}_{\mathrm{dec}}(\mathcal{RNN}_{\mathrm{dyn}}(\{\mathbf{u}_i(\tau)\}_{\tau\in\mathscr{I}^i}, t; \mathbf{w}^*_{\mathrm{dyn}}); \mathbf{w}^*_{\mathrm{dec}})).$$

In what follows, we denote this method as AE/ODE and AE/LSTM, depending on the architecture employed for $\mathcal{RNN}_{\mathrm{dyn}}$.

We remark that the encoder $\mathcal{NN}_{\mathrm{enc}}$ is only instrumental to recover labeled data $\hat{\mathbf{s}}_i(\tau)$ needed to train $\mathcal{RNN}_{\mathrm{dyn}}$ and it is not used to predict the output during the testing phase. In other words, only $\mathcal{RNN}_{\mathrm{dyn}}$ and $\mathcal{NN}_{\mathrm{dec}}$ are retained in the testing phase. The latter observation suggests a third training stage in which, starting from the pre-trained values $(\mathbf{w}^*_{\mathrm{dyn}}, \mathbf{w}^*_{\mathrm{dec}})$, we further train the $\mathcal{RNN}_{\mathrm{dyn}}$ and $\mathcal{NN}_{\mathrm{dec}}$ in a simultaneous manner, that is in an end-to-end (e2e) way:

$$\begin{aligned}(\mathbf{w}^{**}_{\mathrm{dyn}}, \mathbf{w}^{**}_{\mathrm{dec}}) = \underset{\mathbf{w}_{\mathrm{dyn}},\mathbf{w}_{\mathrm{dec}}}{\operatorname{argmin}} \sum_{i\in\mathcal{S}_{\mathrm{train}}} \sum_{t\in\mathscr{T}^i} \|\mathcal{NN}_{\mathrm{dec}}(\mathcal{RNN}_{\mathrm{dyn}}(\{\mathbf{u}_i(\tau)\}_{\tau\in\mathscr{I}^i}, t; \mathbf{w}_{\mathrm{dyn}}); \mathbf{w}_{\mathrm{dec}}) - \mathbf{Y}_i(t)\|^2_{\mathbf{Y}} \\ + \alpha_{\mathrm{dyn}}\mathcal{R}(\mathbf{w}_{\mathrm{dyn}}) + \alpha_{\mathrm{dec}}\mathcal{R}(\mathbf{w}_{\mathrm{dec}})\end{aligned}$$

To denote the models obtained after this third training stage, we will employ the abbreviation AE/ODE-e2e and AE/LSTM-e2e.

2.3 Predicting the evolution without latent dynamics

In Test Case 1c, we compare the results obtained with LDNets with a model consisting of an ODE-Net fed by the query point and by the input signal, that is thus denoted by $(\mathbf{x}, \mathbf{u}(t))$-ODE-Net. This consists in solving the following system of ODEs for each query point $\mathbf{x}$:

$$\begin{cases} \dfrac{\partial \widetilde{\mathbf{y}}(\mathbf{x},t)}{\partial t} = \mathcal{NN}_{\mathrm{dyn}}(\widetilde{\mathbf{y}}(\mathbf{x},t), \mathbf{u}(t), \mathbf{x}; \mathbf{w}_{\mathrm{dyn}}) & \text{in } (0,T] \\ \widetilde{\mathbf{y}}(\mathbf{x},0) = \mathbf{y}(\mathbf{x},0). \end{cases} \tag{4}$$

The results of the comparison are reported in the main text.

3 Results

3.1 Test Case 1: Advection-Diffusion-Reaction equation

In this test case, data are generated through numerical approximation of the PDE model

$$\begin{aligned} &\frac{\partial z(x,t)}{\partial t} - \mu_1 \frac{\partial^2 z(x,t)}{\partial x^2} - \mu_2 \frac{\partial z(x,t)}{\partial x} + \mu_3 z(x,t) = f(x,t) && x \in (-1,1),\, t \in (0,T], \\ &z(-1,t) = z(1,t) && t \in (0,T], \\ &z(x,0) = z_0(x) && x \in (-1,1). \end{aligned} \tag{5}$$

with $f(x,t) = A(t)\cos(2\pi F(t)x - P(t))$, that is a sine wave with amplitude $A(t)$, frequency $F(t)$ and phase $P(t)$. The numerical solver employed to generate training and testing data is described in the main text.

We consider three different cases (Test Case 1a, 1b, 1c), according to whether or not the three parameters (μ_1, μ_2 and μ_3) and the three time-dependent signals ($A(t)$, $F(t)$ and $P(t)$) are considered as fixed or as inputs of the model. In all the test cases, we define y_{norm} as the difference between maximum and minimum value taken by the output on the whole training set. In all the cases presented below, unless otherwise stated, we set $\Delta t = 5 \cdot 10^{-2}$.

3.1.1 Sampling of inputs

In order to generate training and testing data, we need to sample the space of inputs. This calls for a probability distribution on the latter space. For the parameters (μ_1, μ_2 and μ_3), we take for simplicity a uniform distribution on a suitable hypercube. For the time-dependent signals ($A(t)$, $F(t)$ and $P(t)$), instead,

probability distributions on function spaces are needed. For $A(t)$ and $P(t)$, we consider a Gaussian Process distribution [19] with mean μ and with the following covariance kernel

$$K(t_1, t_2) = \sigma^2 \exp\left[-\frac{(t_1 - t_2)^2}{2\tau^2}\right],$$

characterized by standard deviation σ and characteristic time-scale τ. For what concerns $F(t)$ instead, in order to let it vary within a bounded set $(f_{\min}, f_{\max})$, we define it as

$$F(t) = \frac{1}{2}\left[f_{\min} + f_{\max} + (f_{\max} - f_{\min}) \tanh\left(\frac{3}{5}\gamma(t)\right)\right],$$

with $\gamma(t)$ sampled from a Gaussian Process with mean $\mu = 0$, standard deviation $\sigma = 1$ and prescribed characteristic time-scale τ. The values of μ, σ and τ are indicated below for each test case.

3.1.2 Test Case 1a: finite latent dimension, constant parameters

We consider the case $z_0(x) = \cos(\pi x)$ and $f \equiv 0$. The inputs are the constant parameters $\mathbf{u}(t) \equiv (\mu_1, \mu_2, \mu_3)$. To generate training and testing data, we employ a Monte Carlo sampling of the hypercube defined by the bounds $(\mu_1, \mu_2, \mu_3) \in [0, 0.05] \times [-0.1, 0.1] \times [0, 0.01]$. We consider 100 training samples and 500 testing samples. We select the hyperparameters according to the algorithm presented in the main text. The ranges of hyperparameter values used in the tuning algorithm are reported in Tab. 1, in the row *tuning*. The selected values are instead reported in the row *final*.

In Tab. 2 we report the training and testing accuracy metrics obtained by training an LDNet with $d_{\mathbf{s}} = 2$ latent variables. In the table we show the accuracy achieved after 500, 5000 or 50000 epochs of BFGS (in all the cases, we first run 200 epochs of Adam).

3.1.3 Test Case 1b: finite latent dimension, time-dependent inputs

We now consider the case when inputs are time-dependent. We thus fix the values of the parameters to $\mu_1 = 0.05$, $\mu_2 = 0$ and $\mu_3 = 0.002$, and we consider the time-dependent forcing term $f(x, t)$ defined above. In order to keep the intrinsic latent dimension equal to 2, we consider a constant frequency (specifically, $F(t) \equiv 0.5$). Instead, we let the amplitude $A(t)$ and the phase $P(t)$ vary in time, as described in Sec. 3.1.1. Specifically, for $A(t)$ we set $\mu = 2/5$, $\sigma = 2/15$ and $\tau = 1$, while for $P(t)$ we set $\mu = 0$, $\sigma = 4/3$ and $\tau = 1$.

First, we consider 100 training samples and we set $\Delta t = 0.05$. We tune the hyperparameters starting from the ranges indicated in Tab. 1, and we select the optimal values reported in the same table. The LDNets predictions $\widetilde{\mathbf{y}}$ for some test samples are displayed against reference outputs $\mathbf{y}$ in Fig. 1.

Now, we perform two tests. First, by keeping $\Delta t = 0.05$, we vary the number of training samples in the set $\{25, 50, 100, 200, 400\}$. Then, we let Δt vary in the set $\{0.2, 0.1, 0.05, 0.02\}$, by keeping the number of training samples equal to 100. In both the cases, we do not vary the hyperparameters reported in Tab. 1. The results of these two tests are reported in the main text.

Test Case 1b provides an ideal testbed to assess the capabilities of LDNets in discovering a compact representation of the FOM state. The state of (5) indeed evolves on a two-dimensional manifold, being the state fully characterized, at any time t, by two scalars. In other terms, provided that the forcing term is defined as in Test Case 1b, the model (5) has an intrinsic latent dimension equal to 2. At any time t, in fact, $z(\cdot, t)$ is a sine wave with frequency 0.5. Among the infinitely many equivalent parametrization, one is given by the Fourier transform of $z(\cdot, t)$ at frequency 0.5, that is determined by its real and imaginary part, respectively denoted by $Re(\hat{z}(0.5))$ and $Im(\hat{z}(0.5))$.

Our results show that, during the training process of an LDNet, the algorithm discovers a compact representation of the solution field $z(\cdot, t)$, represented by the two latent variables $s_1(t)$ and $s_2(t)$. We now investigate whether there is a relationship between the pairs (s_1, s_2) and $(Re(\hat{z}(0.5)), Im(\hat{z}(0.5)))$. With this goal, we train four different LDNets, starting from a different random initializations of the trainable parameters. Then, we evaluate the trained LDNets on 24 test samples, and we collect the trajectories in the latent space (s_1, s_2). Finally, we plot these trajectories by displaying each point with a color that depends on the corresponding value of $Re(\hat{z}(0.5))$ computed from the reference solution, and we repeat the same procedure by considering the values of $Im(\hat{z}(0.5))$. The results are shown in Fig. 2. We notice that

**Test cases**		**Hyperparameters**						**Trainable parameters**	
		$\mathcal{NN}_{\text{dyn}}$		$\mathcal{NN}_{\text{rec}}$		Δt_{ref}	α_{dyn}, α_{rec}	$\mathcal{NN}_{\text{dyn}}$	$\mathcal{NN}_{\text{rec}}$
		layers	neurons	layers	neurons			# param.	# param.
**Test Case 1a**									
tuning		2	3 – 30	2	3 – 15	$[10^{-1}, 10^{1}]$	0		
final		2	9	2	11	0.5	0	164	188
**Test Case 1b**									
tuning		2	3 – 30	2	3 – 15	$[10^{-1}, 10^{1}]$	$[10^{-6}, 10^{-1}]$		
final		2	10	2	7	2.3	10^{-5}	182	92
**Test Case 1c**									
tuning		1 – 3	4 – 60	1,2	4 – 60	$[10^{-1}, 10^{1}]$	$[10^{-7}, 10^{-1}]$		
fine tuning		2	twice $\mathcal{NN}_{\text{rec}}$	2	4 – 30	8	$[1 \cdot 10^{-7}, 2 \cdot 10^{-3}]$		
$f_{\max}$	$d_{\mathbf{s}}$								
0.5	2	2	10	2	5	8	$1.95 \cdot 10^{-3}$	192	56
0.5	3	2	16	2	8	8	$2.10 \cdot 10^{-4}$	435	121
0.5	5	2	26	2	13	8	$2.00 \cdot 10^{-6}$	1071	287
0.5	7	2	40	2	20	8	$1.14 \cdot 10^{-7}$	2367	621
1	2	2	12	2	6	8	$1.05 \cdot 10^{-3}$	254	73
1	3	2	14	2	7	8	$4.20 \cdot 10^{-4}$	353	99
1	5	2	28	2	14	8	$1.08 \cdot 10^{-6}$	1209	323
1	7	2	52	2	26	8	$1.61 \cdot 10^{-7}$	3699	963
2	2	2	14	2	7	8	$6.60 \cdot 10^{-4}$	324	92
2	3	2	16	2	8	8	$3.90 \cdot 10^{-4}$	435	121
2	5	2	26	2	13	8	$1.23 \cdot 10^{-6}$	1071	287
2	7	2	38	2	19	8	$1.58 \cdot 10^{-6}$	2173	571

Supplementary Table 1. Test Case 1: hyperparameters ranges and selected values. We remark that, in the fine tuning stage of test Case 1c, we select twice as many neurons for $\mathcal{NN}_{\text{dyn}}$ as for $\mathcal{NN}_{\text{rec}}$, in order to reduce the number of independent hyperparameters. See text for details.

BFGS epochs	500	5000	50000
Training time	8m 5s	1h 5m	10h 8m
$\mathrm{NRMSE}_{\mathrm{train}}$	$1.02 \cdot 10^{-3}$	$7.08 \cdot 10^{-5}$	$1.81 \cdot 10^{-5}$
$\mathrm{NRMSE}_{\mathrm{test}}$	$1.19 \cdot 10^{-3}$	$7.23 \cdot 10^{-5}$	$1.88 \cdot 10^{-5}$
$1 - \rho_{\mathrm{train}}$	$9.42 \cdot 10^{-6}$	$4.58 \cdot 10^{-8}$	$3.00 \cdot 10^{-9}$
$1 - \rho_{\mathrm{test}}$	$1.33 \cdot 10^{-5}$	$4.87 \cdot 10^{-8}$	$3.30 \cdot 10^{-9}$

Supplementary Table 2. Test Case 1a: training and test accuracy metrics for LDNets trained with an increasing number of BFGS training epochs (500, 5000 and 50000). Training time refer to a single-CPU standard laptop.

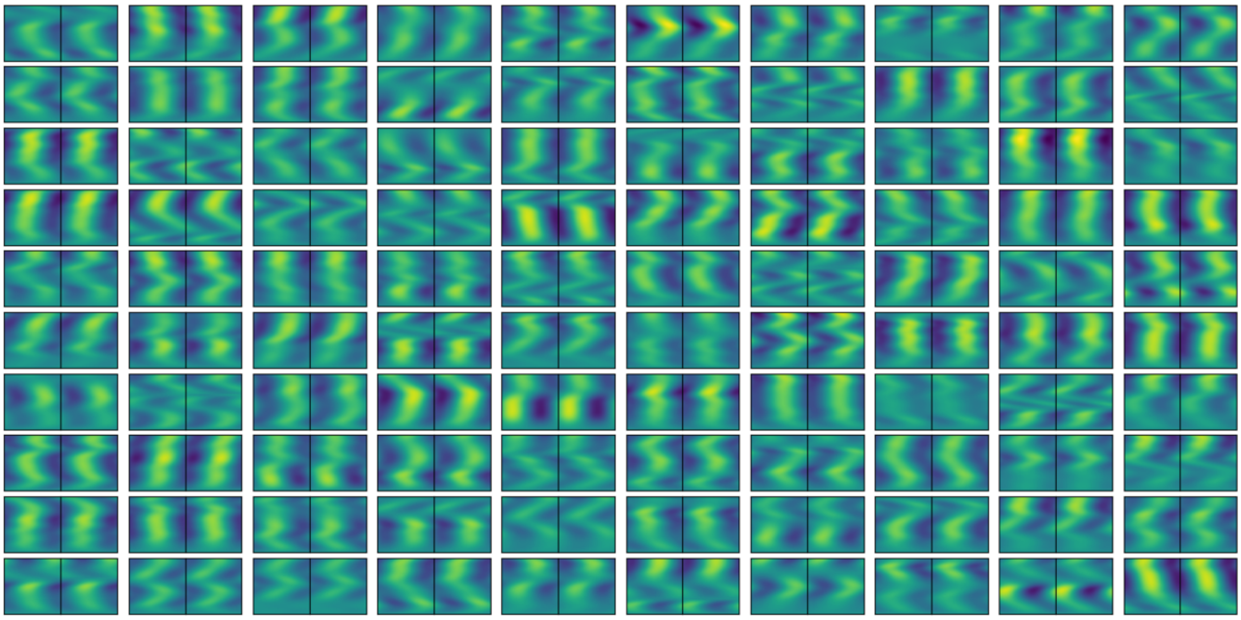

Supplementary Figure 1. Test Case 1b. 100 testing samples, comparing the reference outputs $\mathbf{y}$ (left) with the LDNet predictions $\widetilde{\mathbf{y}}$ (right). For each sample, the horizontal axis refers to space, and the vertical axis refers to time.

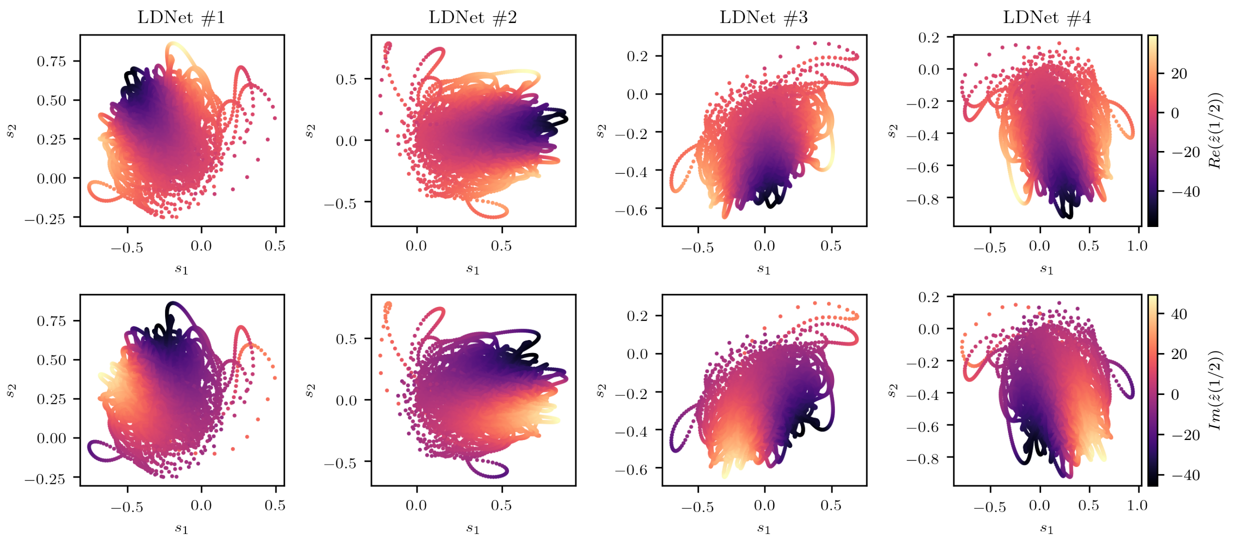

Supplementary Figure 2. Test Case 1b. Trajectories in the latent space (s_1, s_2) of 24 testing samples obtained with four different LDNets, by starting from as many different initial guesses for the trainable parameters (each of them corresponding to different columns). In the first (respectively, second) row, each point in the latent space is colored according to the corresponding value of $Re(\hat{z}(0.5))$ (respectively, $Im(\hat{z}(0.5))$).

trajectories significantly differ among the four LDNets. This is not surprising, as latent states are internal variables in the LDNet, hidden within the input-output relationship. Nonetheless, a common pattern emerges in the connection between the latent states and the Fourier coefficients: each of the four trained LDNets underlies a well-defined relation between (s_1, s_2) and $(Re(\hat{z}(0.5)), Im(\hat{z}(0.5)))$. As a matter of fact, each LDNet discovers a different compact encoding for the FOM state, each of which underlying a relationship with the Fourier coefficients of the solution. In other terms, despite not being explicitly instructed to do that, the LDNet *discovers* an operator that is equivalent to the Fourier transform of the state. At the same time, the reconstruction NN ($\mathcal{NN}_{\text{rec}}$) *discovers* the inverse operator, as it is able to reconstruct the function $z(\cdot, t)$ from the two scalars (s_1, s_2). Remarkably, this is obtained in a fully data-driven manner, without explicitly using any prior Fourier-based feature extraction.

3.1.4 Test Case 1c: infinite latent dimension

As a final step of Test Case 1, we consider the case when the frequency of the forcing term $f(x, t)$ varies in time as well (see Sec. 3.1.1). Specifically, to sample $F(t)$ we set $\tau = 1$, $f_{\min} = 0.25$ and $f_{\max}$ as indicated below; for $A(t)$ we set $\mu = 1$, $\sigma = 1/3$ and $\tau = 1$; for $P(t)$ we set $\mu = 0$, $\sigma = 4/3$ and $\tau = 1$.

In this test case, we are interested in studying the impact of the number of latent states $d_{\mathbf{s}}$ on the LDNet accuracy, in three increasingly challenging cases, namely by setting $f_{\max} = 0.5$, $f_{\max} = 1$ and $f_{\max} = 2$ (see Fig. 3). In all the cases, we consider $d_{\mathbf{s}} \in \{2, 3, 4, 5\}$. For each combination of $f_{\max}$ and $d_{\mathbf{s}}$, we retune the hyperparameters, in order to compare the (in principle) best accuracy attainable. With this purpose, we first run a preliminary hyperparameter tuning step, by considering a wide range of values (see Tab. 1, row *tuning*). Then, we shrink the variability by selecting the most (generally with respect to different combinations) promising area of the hyperparameter space, and we perform a fine tuning for each combination of $f_{\max}$ and $d_{\mathbf{s}}$ independently (row *fine tuning*). The selected hyperparameters are listed in Tab. 1 and the results of this comparison are reported and commented in the main text.

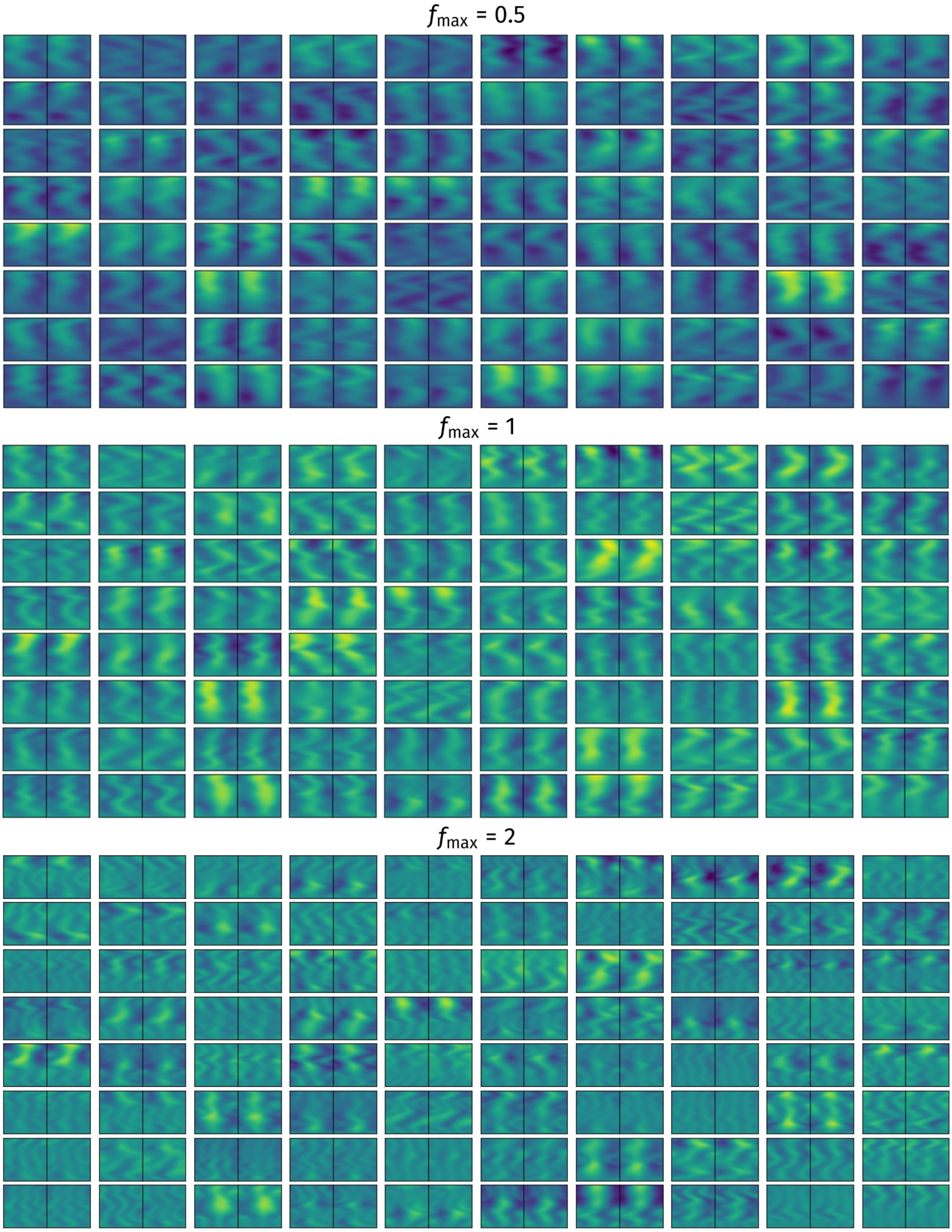

Supplementary Figure 3. Test Case 1c. 80 testing samples for each $f_{\max}$ value considered in this work (namely 0.5, 1 and 2), comparing the reference outputs $\mathbf{y}$ (left) with the LDNet predictions $\widetilde{\mathbf{y}}$ (right), for $d_{\mathbf{s}} = 5$. For each sample, the horizontal axis refers to space, and the vertical axis refers to time.

3.2 Test Case 2: Unsteady Navier-Stokes

In this test case, data are generated through the PDE model

$$\begin{aligned}
&\rho \frac{\partial \mathbf{v}}{\partial t} + \rho\left(\mathbf{v}\cdot\nabla\right)\mathbf{v} - \mu\Delta\mathbf{v} + \nabla p = \mathbf{0} && \mathbf{x}\in\Omega,\, t\in(0,T],\\
&\nabla\cdot\mathbf{v} = 0 && \mathbf{x}\in\Omega,\, t\in(0,T],\\
&\mathbf{v} = u(t)\mathbf{e}_1 && \mathbf{x}\in\Gamma_{\text{top}},\, t\in(0,T],\\
&\mathbf{v} = \mathbf{0} && \mathbf{x}\in\partial\Omega\setminus\Gamma_{\text{top}},\, t\in(0,T],\\
&\mathbf{v} = \mathbf{0} && \mathbf{x}\in\Omega, t=0,
\end{aligned} \tag{6}$$

that is the Navier-Stokes equations, where $\mathbf{v}$ represents the velocity field while p defines the pressure field. We consider a variation of the 2D lid-driven cavity benchmark in which the velocity prescribed on the top portion of the boundary (Γ_{top}) is a time-dependent input $u(t)$. In particular, as reported in Sec. 3.1.1, we consider a Gaussian Process Distribution with mean $\mu = 0$, standard deviation $\sigma = 5$ and characteristic time-scale $\tau = 5$.

The numerical solver employed to generate training, validation and testing data is described in the main text. The training and validation datasets consist of 80 simulations with $T = 20$, where 200 points are uniformly sampled in $\Omega = (0,1)^2$. Regarding the testing data, we run 200 simulations with $T = 40$, a time span that is twice as long as the one of the training set, and we uniformly take 400 points in space from the domain $\Omega = (0,1)^2$. We consider a semi-implicit time discretization with $\Delta t = 2\cdot 10^{-1}$. We employ Taylor-Hood elements in the FEM solver, i.e. $\mathbb{P}_2$ Finite Elements for $\mathbf{v}$ and $\mathbb{P}_1$ Finite Elements for p.

We fix the number of latent states to 1, 5 and 10 respectively and we perform hyperparameter tuning by monitoring the following goal-oriented loss function:

$$\mathcal{E}(\mathbf{v},\hat{\mathbf{v}}) = \frac{\|\mathbf{v}-\hat{\mathbf{v}}\|^2}{v_{\text{norm}}^2} + \gamma\left\|\frac{\mathbf{v}}{\epsilon+\|\mathbf{v}\|} - \frac{\hat{\mathbf{v}}}{\epsilon+\|\hat{\mathbf{v}}\|}\right\|^2. \tag{7}$$

Specifically, we pick $\gamma = 1\cdot 10^{-1}$ and $\epsilon = 1\cdot 10^{-4}$. Due to the presence of long tails in the velocity distribution, in this test case we employ the compressing layer in $\mathcal{NN}_{\text{dec}}$ (see Methods).

We define in Tab. 3 the initial ranges and final values of the hyperparameters for different numbers of latent states (1, 5 and 10).

We report in Tab. 4 the NRMSE and Pearson dissimilarity values on the test set for $d_{\mathbf{s}} = 1$, $d_{\mathbf{s}} = 5$ and $d_{\mathbf{s}} = 10$. We notice that the size of both $\mathcal{NN}_{\text{dyn}}$ and $\mathcal{NN}_{\text{rec}}$ architectures increases while the test error of the LDNets decreases with respect to the number of latent states. Similarly to Test Case 1, as the number of latent states increases the LDNets are more and more efficient in discovering an effective compact representation of the system state and thus providing reliable predictions. Still, small NRMSEs are attained even with a small number of latent states.

We test the trained LDNets also in a time-extrapolation regime, that is for $t \in (20, 40]$, when the training samples are generated for $t \in [0, 20]$. As reported in Tab. 4, the test errors obtained in extrapolation are not significantly different than the ones obtained in the same time horizon seen during training. This is a remarkable achievement, considering that the dynamical system at hand does not present a periodic or quasi-periodic regime.

For the sake of completeness, we depict in Fig. 4 the time evolution of the streamlines associated with a specific sample belonging to the test set, along with the corresponding input signal $u(t)$. We see that the approximation provided by LDNets with 10 latent variables has an excellent agreement with the FOM.

Moreover, we report in Fig. 5 the time evolution of the latent variables in 6 different test samples belonging to the test set. It is interesting to observe that, in the case $d_{\mathbf{s}} = 1$, the unique latent variable has similar trajectories to the input $u(t)$, even if with some delay, thus capturing the dynamics of the main vortices. When additional latent variables are introduced, we observe trajectories with different shapes, which allows to capture the dynamics of smaller structures in the fluid velocity field, as testified by the reduced errors of Tab. 4.

**Test cases**	**Hyperparameters**						**Trainable parameters**	
	$\mathcal{NN}_{\text{dyn}}$		$\mathcal{NN}_{\text{rec}}$		Δt_{ref}	α_{dyn}, α_{rec}	$\mathcal{NN}_{\text{dyn}}$	$\mathcal{NN}_{\text{rec}}$
	layers	neurons	layers	neurons			# param.	# param.
**Test Case** $d_{\mathbf{s}} = 1$								
tuning	1 – 6	5 – 35	1 – 6	5 – 35	$[10^{-1}, 10^{1}]$	0		
final	2	7	4	24	5.4	0	85	1'970
**Test Case** $d_{\mathbf{s}} = 5$								
tuning	1 – 6	5 – 35	1 – 6	5 – 35	$[10^{-1}, 10^{1}]$	0		
final	2	27	4	33	8.6	0	1'085	3'731
**Test Case** $d_{\mathbf{s}} = 10$								
tuning	1 – 6	5 – 35	1 – 6	5 – 35	$[10^{-1}, 10^{1}]$	0		
final	3	19	5	28	9.2	0	1'188	3'698

Supplementary Table 3. Test Case 2: hyperparameters ranges and selected values. See text for details.

Number of latent states	1	5	10
$\text{NRMSE}_{\text{test}}$ $(0 < t < 40)$	$4.08 \cdot 10^{-3}$	$1.94 \cdot 10^{-3}$	$1.39 \cdot 10^{-3}$
$\text{NRMSE}_{\text{test}}$ $(0 < t < 20)$	$4.10 \cdot 10^{-3}$	$1.93 \cdot 10^{-3}$	$1.34 \cdot 10^{-3}$
$\text{NRMSE}_{\text{test}}$ $(20 < t < 40)$	$4.07 \cdot 10^{-3}$	$1.95 \cdot 10^{-3}$	$1.44 \cdot 10^{-3}$
$1 - \rho_{\text{test}}$ $(0 < t < 40)$	$5.14 \cdot 10^{-3}$	$1.16 \cdot 10^{-3}$	$6.01 \cdot 10^{-4}$
$1 - \rho_{\text{test}}$ $(0 < t < 20)$	$5.48 \cdot 10^{-3}$	$1.23 \cdot 10^{-3}$	$6.00 \cdot 10^{-4}$
$1 - \rho_{\text{test}}$ $(20 < t < 40)$	$4.81 \cdot 10^{-3}$	$1.11 \cdot 10^{-3}$	$6.02 \cdot 10^{-4}$

Supplementary Table 4. Test Case 2: test accuracy metrics for LDNets trained with an increasing number of latent states (1, 5 and 10).

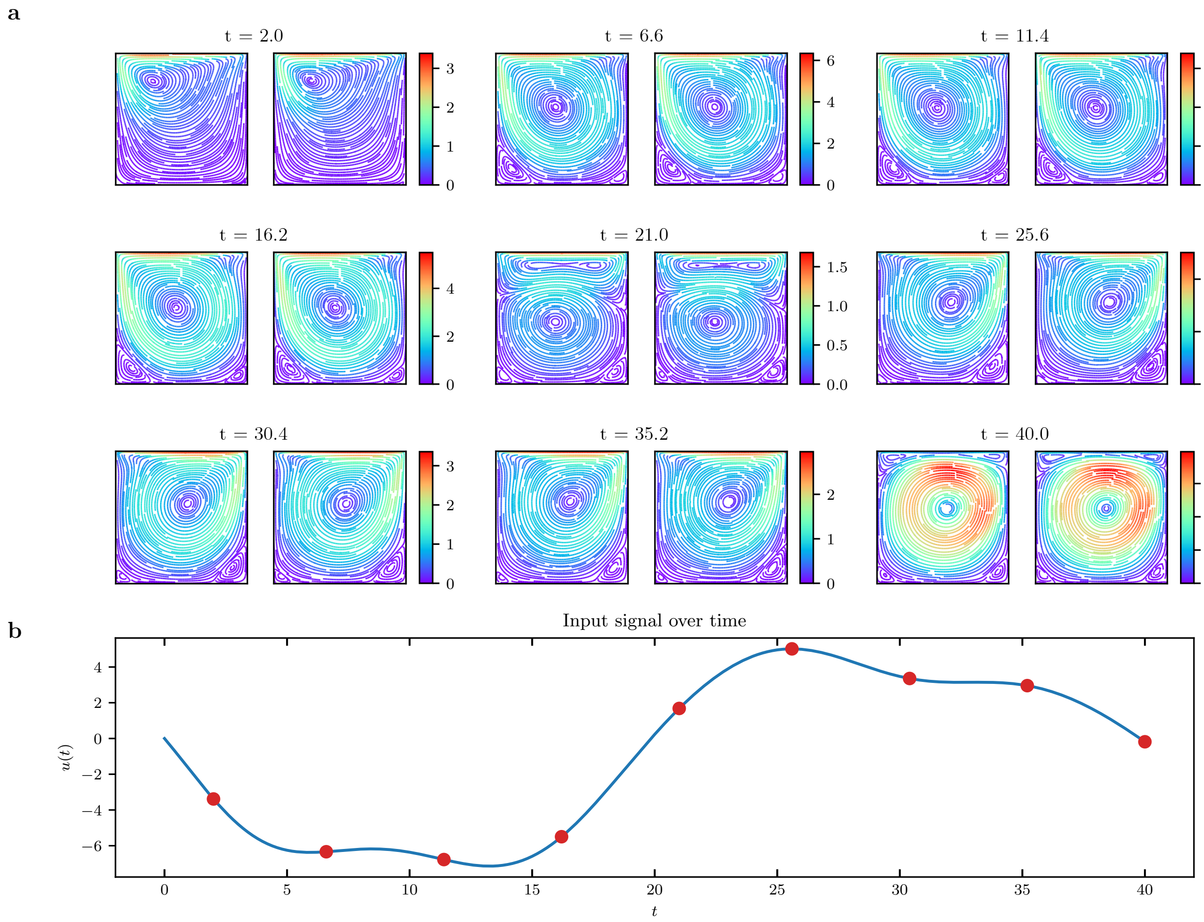

Supplementary Figure 4. Test Case 2. **(a)**: Streamlines of the velocity field $\mathbf{v}$ for one testing sample over time ($t \in [0, 40]$). Horizontal and vertical axis refer to space in the domain $\Omega = (0,1)^2$. For each subplot, the left one represents the FOM while the right one represents the LDNet approximation for $d_{\mathbf{s}} = 10$. **(b)**: Input signal $u(t)$ over time applied to the top portion of the boundary Γ_{top}.

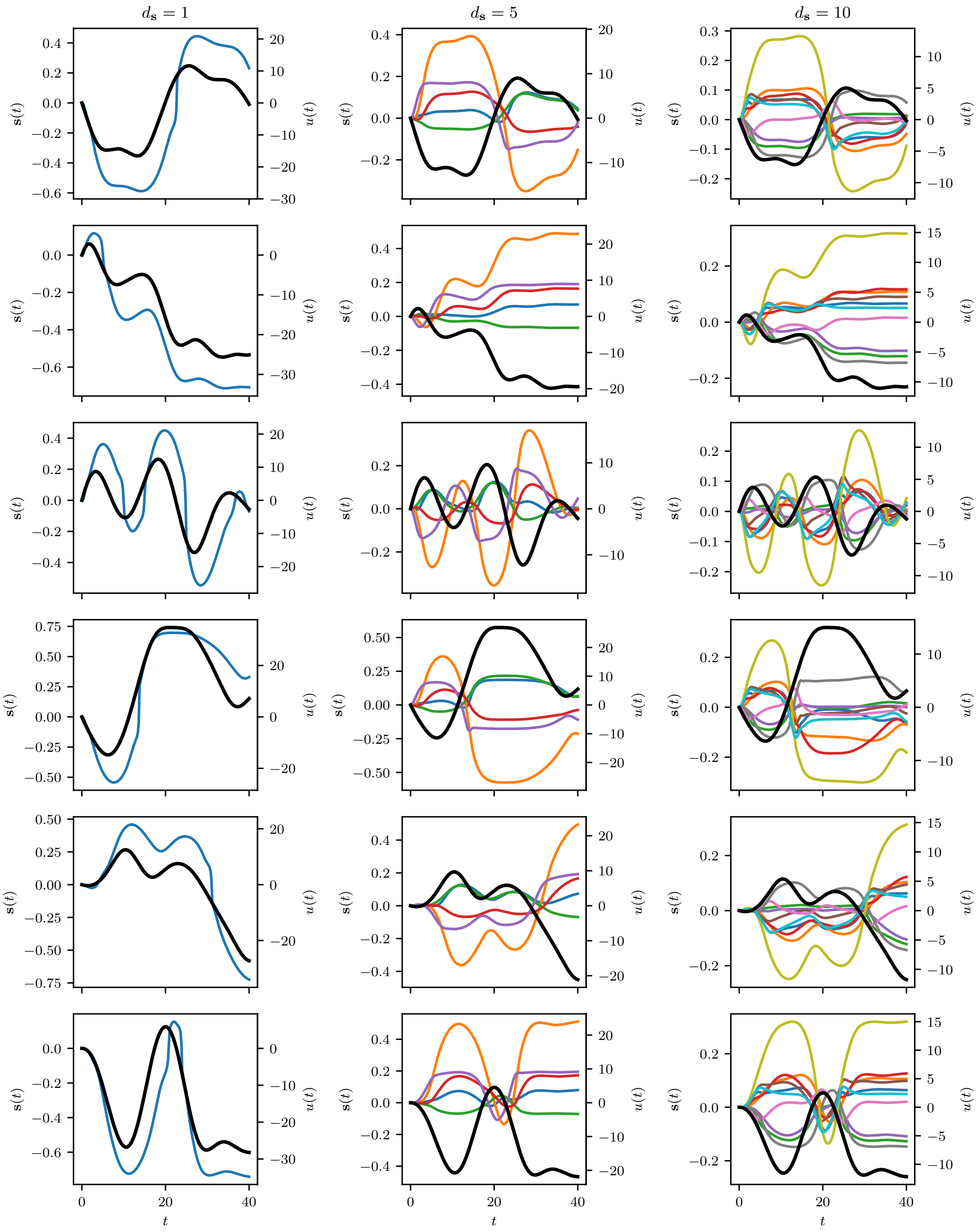

Supplementary Figure 5. Test Case 2. Evolution of the latent variables in 6 different samples belonging to the test set (one for each row), for the three trained models – with 1, 5 and 10 latent variables respectively – considered in this test case (one model for each column). In each plot we display in colored thin lines the latent variables $\mathbf{s}(t)$ (ticks on left axis), and in black thick line the associated input $u(t)$ (ticks on right axis).

	Hyperparameters							
	$d_{\mathbf{s}}$	$\mathcal{NN}_{\text{enc}}$, $\mathcal{NN}_{\text{dec}}$		$\mathcal{NN}_{\text{dyn}}$		Δt_{ref}	α_{enc}, α_{dec}	α_{dyn}
		layers	neurons	layers	neurons			
**AE/ODE**								
tuning	4 – 12	1 – 4	4 – 80	1 – 10	4 – 40	$[10^1, 10^3]$	$[10^{-5}, 10^{-2}]$	$[10^{-5}, 10^{-1}]$
final	12	1	75	4	38	$1.80 \cdot 10^1$	$6.89 \cdot 10^{-3}$	$2.80 \cdot 10^{-2}$
**AE/LSTM**								
tuning	4 – 12	1 – 4	4 – 80				$[10^{-5}, 10^{-2}]$	$[10^{-5}, 10^0]$
final	12	1	75				$6.89 \cdot 10^{-3}$	$4.81 \cdot 10^{-1}$

Supplementary Table 5. Test Case 3: hyperparameters ranges and selected values for the AE/ODE and AE/LSTM models. For the encoder (respectively, the decoder) the number of neurons refers to the first (respectively, last) hidden layer. In the other layers, the number of neurons is linearly varied to connect the first (respectively, last) hidden layer to the layer of latent variables.

3.3 Test Case 3: 1D electrophysiology model

We consider the Monodomain model coupled with the Aliev-Panfilov ionic model [1]:

$$\begin{aligned}
&\frac{\partial z}{\partial t} - D\frac{\partial^2 z}{\partial x^2} = Kz(1-z)(z-\alpha) - zw + I_{\text{stim}} && x \in (0, L),\, t \in (0, T], \\
&\frac{\partial w}{\partial t} = \left(\gamma + \frac{\mu_1 w}{\mu_2 + z}\right)(-w - Kz(z-b-1)) && x \in (0, L),\, t \in (0, T], \\
&\frac{\partial z(0,t)}{\partial x} = \frac{\partial z(L,t)}{\partial x} = 0 && t \in (0, T], \\
&z(x,0) = w(x,0) = 0, && x \in (0, L).
\end{aligned} \tag{8}$$

with parameters $D = 0.1\,\text{mm}^2\,\text{ms}^{-1}$, $K = 8$, $\alpha = 0.1$, $\gamma = 0.02$, $\mu_1 = 0.2$, $\mu_2 = 0.3$, $b = 0.15$, $L = 100\,\text{mm}$, $T = 500\,\text{ms}$. Note that z is a non-dimensional potential (which can be mapped to its physiological values by the relationship $(100z - 80)$mV) and the model is rescaled with respect to the time constant $\tau = 12.9\,\text{ms}$ (for further details, see [11]).

We consider two stimulation points, namely $x_1^{\text{stim}} = L/4$ and $x_2^{\text{stim}} = 3L/4$. The input of the model is given by $\mathbf{u}(t) = (I_{\text{stim}}(x_1^{\text{stim}}, t), I_{\text{stim}}(x_2^{\text{stim}}, t))$. To generate the training samples, we randomly trigger the applied stimuli, either in correspondence of x_1^{stim}, x_2^{stim} or both points, by randomly picking the stimulation times. We consider 100 training samples and 100 testing samples. In this test case, we aim to predict the evolution of the scalar field $z(x, t)$. For the approximation of the AP model, we employ the finite difference method both in space and time, on a regular grid with 800 points in space and 10^5 time steps. Then, we subsample the space-time grid by retaining 100 points in space and 500 time instants.

We compare the results obtained with several methods by employing the same training and testing data. Specifically, we consider the POD-DEIM method, auto-encoder-based methods (AE/ODE, AE/ODE-e2e, AE/LSTM, AE/LSTM-e2e) and LDNets. In order to ensure a fair comparison, for all the methods that require a choice of hyperparameters we use an algorithm for their automatic tuning, as described in the main text. The ranges used for tuning and the final hyperparameter values are reported in Tab. 5 and 6. In this test case, we employ the technique described in the main text to impose in a strong manner the equilibrium condition of the initial state when $\mathbf{u}(t) = \mathbf{0}$. To achieve a significant dimensionality reduction, we set a maximum number of 12 latent variables both for auto-encoder-based methods and for LDNets. We notice that the hyperparameter tuning algorithm selects the maximum number of latent states (i.e. $d_{\mathbf{s}} = 12$) for all the methods. For the POD-DEIM method, we test different number $d_{\mathbf{s}}$ of POD modes (i.e., basis functions) for the state and the nonlinear term, ranging from 12 to 60.

We observe that the results obtained with the POD-DEIM method using 12 modes are unsatisfactory, compared to the other methods, confirming the importance of adopting nonlinear dimensional reduction techniques for this class of traveling-front problems. Then, we consider higher number of modes, namely 24,

	Hyperparameters						
	$d_{\mathbf{s}}$	$\mathcal{NN}_{\text{dyn}}$		$\mathcal{NN}_{\text{rec}}$		Δt_{ref}	α_{dyn}, α_{rec}
		layers	neurons	layers	neurons		
**LDNet**							
tuning	4 – 12	1 – 3	4 – 15	1 – 5	4 – 20	$[10^1, 10^3]$	$[10^{-5}, 10^{-2}]$
final	12	1	8	5	17	$2.05 \cdot 10^2$	$4.70 \cdot 10^{-3}$

Supplementary Table 6. Test Case 3: hyperparameters ranges and selected values for the LDNet.

36, 48 and 60. We report in Fig. 6 and Fig. 7 the results. By increasing the number of considered modes, the accuracy increases. With 60 modes, e.g., the POD-DEIM method is more accurate than auto-encoder-based methods with 12 latent variables, while LDNets with 12 latent variables are still more accurate.

We observe that, in this test case, the latent variables of the LDNet diverge as time goes by, as the system under consideration is also characterized by divergent dynamics, associated with waves propagating from their sources, with no return to the resting state. In constrast, in Test Case 2, wherein we consider time horizons long enough to contain the reversal in the flow directions, we observe latent variables that often return close to the origin (see Fig. 5).

3.4 Test Case 4: 2D electrophysiology model with reentrant activity

We consider a two-dimensional isotropic Monodomain equation coupled with the Aliev-Panfilov ionic model [1, 9]:

$$\begin{aligned}
&\frac{\partial z}{\partial t} - \nabla \cdot (D \nabla z) = K z (1-z)(z-\alpha) - z w + I_{\text{stim}} && \mathbf{x} \in \Omega,\ t \in (0,T], \\
&\frac{\partial w}{\partial t} = \left(\gamma + \frac{\mu_1 w}{\mu_2 + z}\right)(-w - K z (z - b - 1)) && \mathbf{x} \in \Omega,\ t \in (0,T], \\
&\nabla z(\mathbf{x},t) \cdot \mathbf{n}(\mathbf{x}) = 0 && \mathbf{x} \in \partial\Omega,\ t \in (0,T], \\
&z(\mathbf{x},0) = w(x,0) = 0, && \mathbf{x} \in \Omega.
\end{aligned} \tag{9}$$

in a square domain Ω with 100 mm long edges and parameters $D = 0.2\,\text{mm}^2\,\text{ms}^{-1}$, $K = 8$, $\alpha = 0.05$, $\gamma = 0.01$, $\mu_1 = 0.2$, $\mu_2 = 0.3$, $b = 0.15$, $T = 450\,\text{ms}$. As done in the previous test case, the solution is non-dimensional and the model is rescaled with respect to the time constant τ.

After a first rightward propagating wavefront, we consider a second circular stimulus

$$I_{\text{stim}}(\mathbf{x}, t; r, t_{\text{app}}) = I_s(t; t_{\text{app}}) \mathbb{I}_{\|\mathbf{x} - \mathbf{x}_c\| < R},$$

applied at the center $\mathbf{x}_c = (50\,\text{mm}, 50\,\text{mm})^T$ of the square domain with radius r, starting at t_{app} and with duration 4 ms. All simulations are initialized 450 ms after the beginning of the first wavefront. The training dataset is constructed by sampling the radius in $[1, 10]$ mm and the second impulse starting times t_{app} in $[65, 87.5]$ ms. The input of the model is thus given by $\mathbf{u}(t) = (r, I_s(t; t_{\text{app}}))$. We consider 200 training/validation samples and 75 test samples, to verify the ability of the models to predict the evolution of the scalar field $z(\mathbf{x}, t)$. For the numerical approximation of (9), we employ P1 finite element method on a structured grid with element size $h = 0.5\,\text{mm}$ and a semi-implicit time advancing scheme, based on a subdivision of the time domain with a time step $\Delta t = 0.25\,\text{ms}$. To generate the datasets, we subsample the space-time grid by retaining 2694 points in space and 180 time instants. When training the LDNet we randomly retain only 20 points for each time instant.

The ranges considered for tuning the hyperparameter and the corresponding selected values are reported in Tabs. 7–8. The results are reported in the main text.

Supplementary References

[1] R. R. Aliev and A. V. Panfilov. “A simple two-variable model of cardiac excitation”. In: *Chaos, Solitons & Fractals* 7.3 (1996), pp. 293–301.

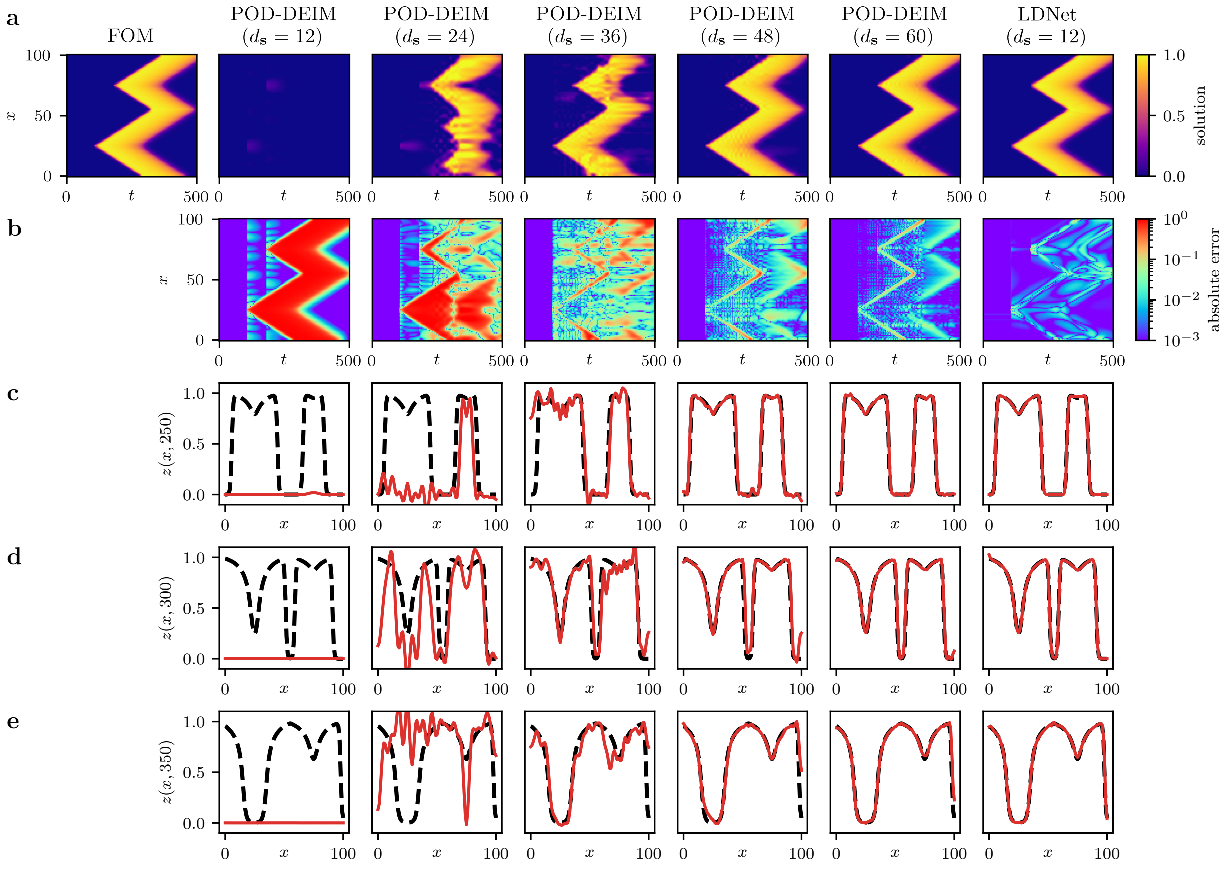

Supplementary Figure 6. We compare the results obtained with the POD-DEIM method, for an increasing number of considered modes (reported in the titles), against the results obtained with our proposed method. The figure shows the predictions obtained for a sample belonging to the test dataset. The left-most column reports the FOM solution of the AP model (the abscissa denotes time, the ordinate denotes space). **(a)** the space-time solution; **(b)** the space-time error with respect to the FOM solution; **(c)-(d)-(e)** three snapshots of the space-dependent output field at $t = 250$, 300 and 350, in which we compare the predicted solution (red solid line) with the FOM solution (black dashed line). For an animated version of this figure, see Supplementary Movies 20–31.

	**Hyperparameters**						
	$\mathcal{NN}_{\text{enc}}, \mathcal{NN}_{\text{dec}}$		$\mathcal{NN}_{\text{dyn}}$		Δt_{ref}	$\alpha_{\text{enc}}, \alpha_{\text{dec}}$	α_{dyn}
	layers	neurons	layers	neurons			
**AE/ODE**							
tuning	$1-4$	$50-250$	$1-3$	$5-24$	$[10^{-2}, 10^{1}]$	$[10^{-5}, 10^{-1}]$	$[10^{-6}, 10^{-1}]$
final	1	220	3	20	$1.76 \cdot 10^{-2}$	$1.98 \cdot 10^{-4}$	$7.28 \cdot 10^{-5}$

Supplementary Table 7. Test Case 4: hyperparameters ranges and selected values for the AE/ODE model. For the encoder (respectively, the decoder) the number of neurons refers to the first (respectively, last) hidden layer. In the other layers, the number of neurons is linearly varied to connect the first (respectively, last) hidden layer to the layer of latent variables.

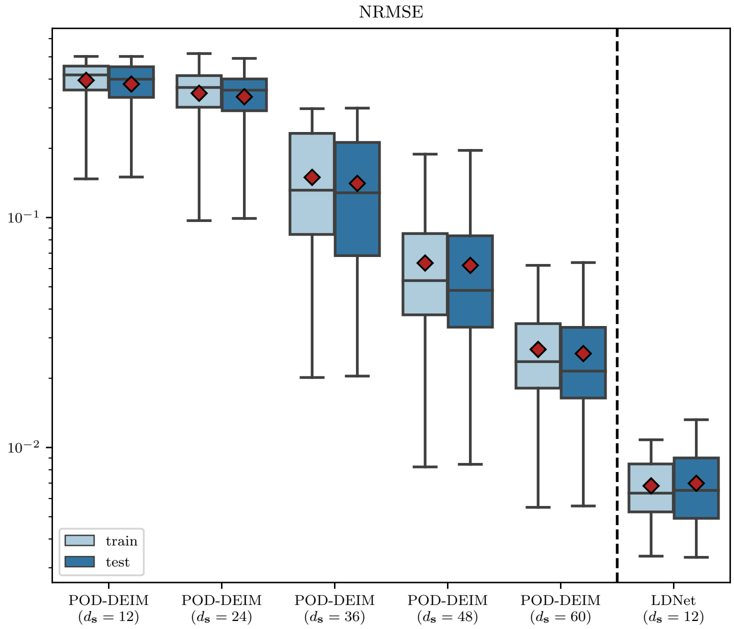

Supplementary Figure 7. We compare the results obtained with the POD-DEIM method, for an increasing number of considered modes, against the results obtained with our proposed method. Specifically, we report boxplots of the distribution of the testing (blue) and training (light blue) errors obtained with each method. The boxes show the quartiles while the whiskers extend to show the rest of the distribution. The red diamonds represent the average error on each dataset.

	**Hyperparameters**					
	$\mathcal{NN}_{\text{dyn}}$		$\mathcal{NN}_{\text{rec}}$		Δt_{ref}	α_{dyn}, α_{rec}
	layers	neurons	layers	neurons		
**LDNet**						
tuning	1 – 3	5 – 24	2 – 4	5 – 29	$[10^{-2}, 10^{1}]$	$[10^{-6}, 10^{-2}]$
final	1	24	4	25	$1.63 \cdot 10^{-1}$	$1.30 \cdot 10^{-3}$

Supplementary Table 8. Test Case 4: hyperparameters ranges and selected values for the LDNet.

[2] A. C. Antoulas. *Approximation of large-scale dynamical systems.* Vol. 6. Siam, 2005.

[3] P. Benner, S. Grivet-Talocia, A. Quarteroni, G. Rozza, W. Schilders, and L. M. Silveira. *Model Order Reduction - Volume 2: Snapshot-Based Methods and Algorithms.* Berlin, Boston: De Gruyter, 2021.

[4] P. Benner, S. Gugercin, and K. Willcox. "A survey of projection-based model reduction methods for parametric dynamical systems". In: *SIAM Review* 57.4 (2015), pp. 483–531.

[5] P. Benner, V. Mehrmann, and D. C. Sorensen. *Dimension reduction of large-scale systems.* Vol. 35. Springer, 2005.

[6] S. L. Brunton, B. R. Noack, and P. Koumoutsakos. "Machine learning for fluid mechanics". In: *Annual review of fluid mechanics* 52 (2020), pp. 477–508.

[7] S. Chaturantabut and D. C. Sorensen. "Nonlinear model reduction via discrete empirical interpolation". In: *SIAM Journal on Scientific Computing* 32.5 (2010), pp. 2737–2764.

[8] R. T. Chen, Y. Rubanova, J. Bettencourt, and D. K. Duvenaud. "Neural ordinary differential equations". In: *Advances in neural information processing systems (NeurIPS 2018 Proceedings)* 31 (2018).

[9] P. C. Franzone, L. F. Pavarino, and S. Scacchi. *Mathematical cardiac electrophysiology.* Vol. 13. Springer, 2014.

[10] S. Fresca, L. Dede', and A. Manzoni. "A comprehensive deep learning-based approach to reduced order modeling of nonlinear time-dependent parametrized PDEs". In: *Journal of Scientific Computing* 87.2 (2021), pp. 1–36.

[11] S. Göktepe and E. Kuhl. "Computational modeling of cardiac electrophysiology: a novel finite element approach". In: *International journal for numerical methods in engineering* 79.2 (2009), pp. 156–178.

[12] J. S. Hesthaven, C. Pagliantini, and G. Rozza. "Reduced basis methods for time-dependent problems". In: *Acta Numerica* 31 (2022), pp. 265–345.

[13] S. Hochreiter and J. Schmidhuber. "Long short-term memory". In: *Neural Computation* 9.8 (1997), pp. 1735–1780.

[14] K. Lee and K. T. Carlberg. "Model reduction of dynamical systems on nonlinear manifolds using deep convolutional autoencoders". In: *Journal of Computational Physics* 404 (2020), p. 108973.

[15] R. Maulik, B. Lusch, and P. Balaprakash. "Reduced-order modeling of advection-dominated systems with recurrent neural networks and convolutional autoencoders". In: *Physics of Fluids* 33.3 (2021), p. 037106.

[16] V. Oommen, K. Shukla, S. Goswami, R. Dingreville, and G. E. Karniadakis. "Learning two-phase microstructure evolution using neural operators and autoencoder architectures". In: *arXiv preprint arXiv:2204.07230* (2022).

[17] C. Prud'Homme, D. V. Rovas, K. Veroy, L. Machiels, Y. Maday, A. T. Patera, and G. Turinici. "Reliable real-time solution of parametrized partial differential equations: Reduced-basis output bound methods". In: *Journal of Fluids Engineering* 124.1 (2002), pp. 70–80.

[18] A. Quarteroni, A. Manzoni, and F. Negri. *Reduced basis methods for partial differential equations: an introduction.* Vol. 92. Springer, 2015.

[19] C. E. Rasmussen. "Gaussian processes in machine learning". In: *Summer school on machine learning.* Springer. 2003, pp. 63–71.

[20] P. R. Vlachas, G. Arampatzis, C. Uhler, and P. Koumoutsakos. "Multiscale simulations of complex systems by learning their effective dynamics". In: *Nature Machine Intelligence* 4.4 (2022), pp. 359–366.
