# Supplementary material for: Learning the intrinsic dynamics of spatio-temporal processes through Latent Dynamics Networks
Source: Nat Commun. 2024 Feb 28;15:1834. doi: 10.1038/s41467-024-45323-x (PMC11258335; doi:10.1038/s41467-024-45323-x)
Supplement: Supplementary file 3 — Description of Additional Supplementary Files [file 41467_2024_45323_MOESM3_ESM.pdf]

Description of Additional Supplementary Files

Supplementary Movies

Supplementary Movie 1: animated versions of Figure 4 of the main text, for sample number 1 of the testing set.

Supplementary Movie 2: animated versions of Figure 4 of the main text, for sample number 2 of the testing set.

Supplementary Movie 3: animated versions of Figure 4 of the main text, for sample number 3 of the testing set.

-**Supplementary Movie** 4: animated versions of Figure 4 of the main text, for sample number 4 of the testing set.

Supplementary Movie 5: animated versions of Figure 4 of the main text, for sample number 5 of the testing set.

Supplementary Movie 6: animated versions of Figure 4 of the main text, for sample number 6 of the testing set.

Supplementary Movie 7: animated versions of Figure 4 of the main text, for sample number 7 of the testing set.

Supplementary Movie 8: animated versions of Figure 4 of the main text, for sample number 8 of the testing set.

Supplementary Movie 9: animated versions of Figure 4 of the main text, for sample number 9 of the testing set.

Supplementary Movie 10: animated versions of Figure 4 of the main text, for sample number 10 of the testing set.

Supplementary Movie 11: animated versions of Figure 5 of the main text, for sample number 1 of the testing set.

Supplementary Movie 12: animated versions of Figure 5 of the main text, for sample number 2 of the testing set.

Supplementary Movie 13: animated versions of Figure 5 of the main text, for sample number 3 of the testing set.

Supplementary Movie 14: animated versions of Figure 5 of the main text, for sample number 4 of the testing set.

Supplementary Movie 15: animated versions of Figure 5 of the main text, for sample number 5 of the testing set.

Supplementary Movie 16: animated versions of Figure 5 of the main text, for sample number 6 of the testing set.

Supplementary Movie 17: animated versions of Figure 5 of the main text, for sample number 7 of the testing set.

Supplementary Movie 18: animated versions of Figure 5 of the main text, for sample number 8 of the testing set.

Supplementary Movie 19: animated versions of Figure 5 of the main text, for sample number 9 of the testing set. - **Supplementary Movie** 20: animated versions of Figure 5 of the main text, for sample number 10 of the testing set.

Supplementary Movie 20: animated versions of Figure 5 of the main text, for sample number 10 of the testing set.

Supplementary Movie 21: animated versions of Figure 6 of the SI, for sample number 1 of the testing set. - **Supplementary Movie** 22: animated versions of Figure 6 of the SI, for sample number 2 of the testing set.

Supplementary Movie 23: animated versions of Figure 6 of the SI, for sample number 3 of the testing set.

Supplementary Movie 24: animated versions of Figure 6 of the SI, for sample number 4 of the testing set.

Supplementary Movie 25: animated versions of Figure 6 of the SI, for sample number 5 of the testing set.

Supplementary Movie 26: animated versions of Figure 6 of the SI, for sample number 6 of the testing set.

Supplementary Movie 27: animated versions of Figure 6 of the SI, for sample number 7 of the testing set.

Supplementary Movie 28: animated versions of Figure 6 of the SI, for sample number 8 of the testing set.

Supplementary Movie 29: animated versions of Figure 6 of the SI, for sample number 9 of the testing set.

Supplementary Movie 30: animated versions of Figure 6 of the SI, for sample number 10 of the testing set.

Supplementary Movie 31: animated versions of Figure 8 of the main text, for 3 different samples of the testing set.
