# Supplementary figures and images for: Learning the intrinsic dynamics of spatio-temporal processes through Latent Dynamics Networks
Source: Nat Commun. 2024 Feb 28;15:1834. doi: 10.1038/s41467-024-45323-x (PMC11258335; doi:10.1038/s41467-024-45323-x)

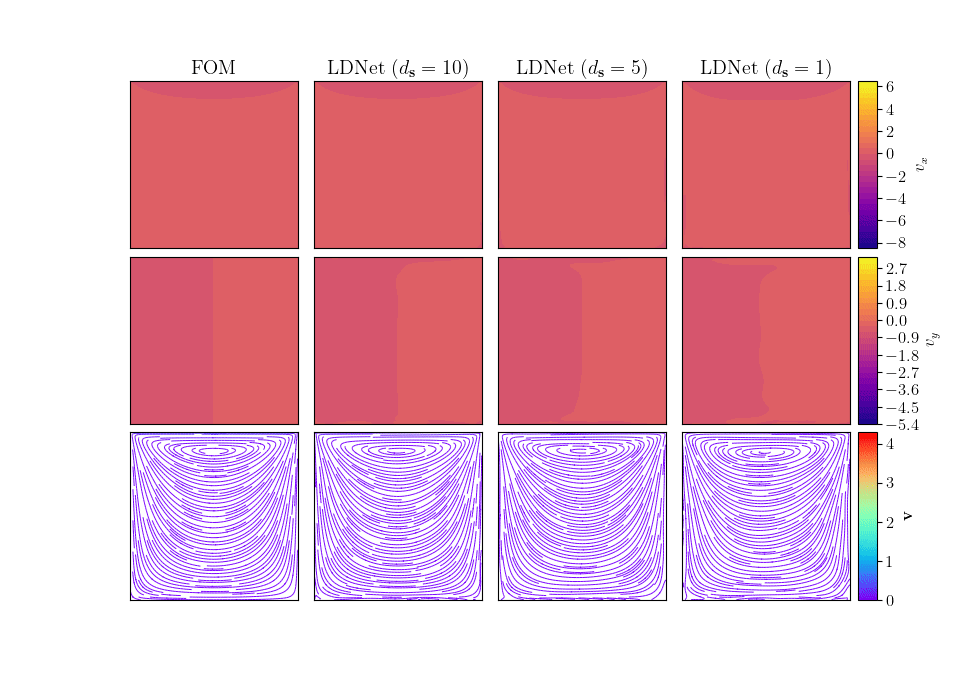

Supplement: Supplementary file 4 — Supplementary Movie 1 [file 41467_2024_45323_MOESM4_ESM.gif]

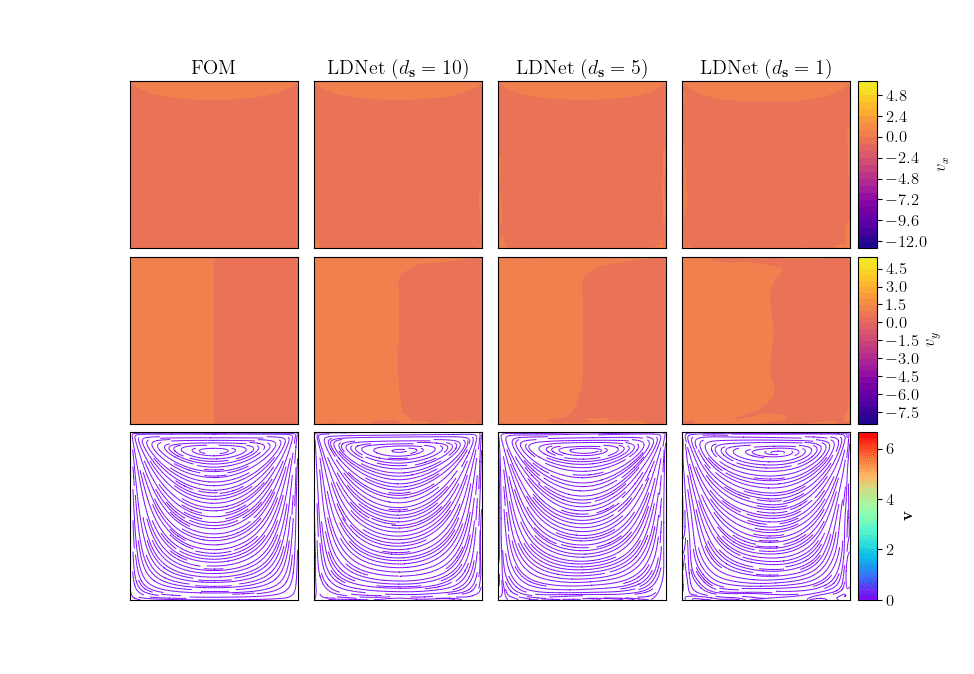

Supplement: Supplementary file 5 — Supplementary Movie 2 [file 41467_2024_45323_MOESM5_ESM.gif]

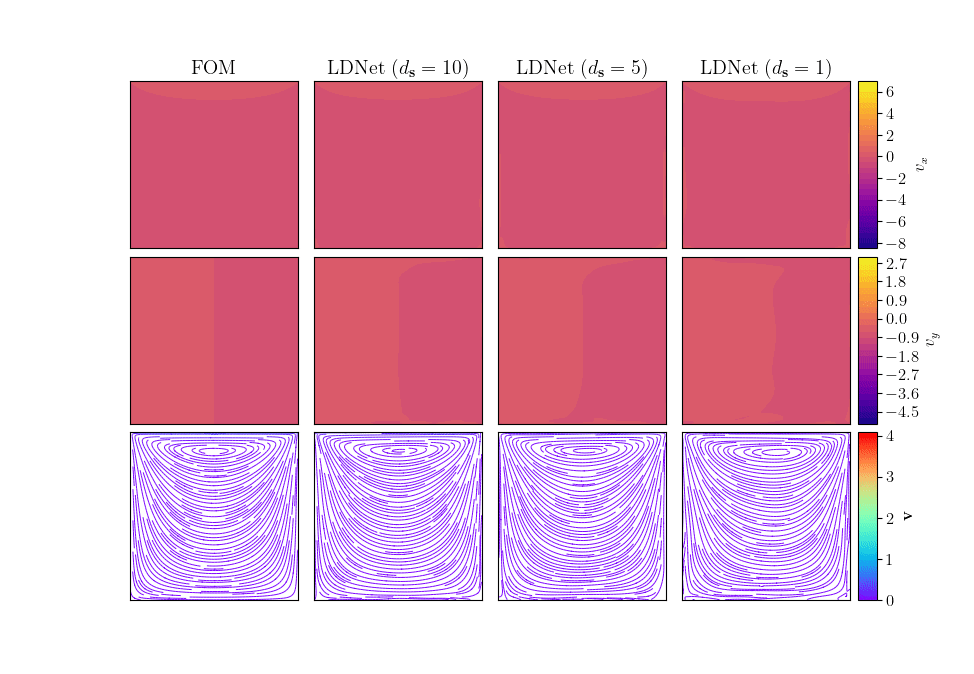

Supplement: Supplementary file 6 — Supplementary Movie 3 [file 41467_2024_45323_MOESM6_ESM.gif]

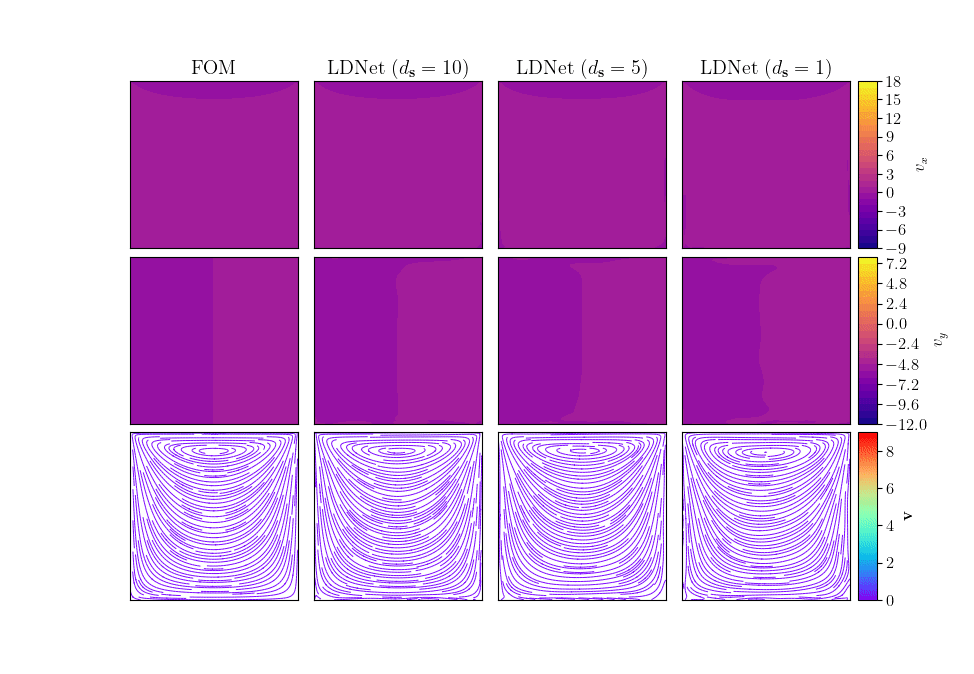

Supplement: Supplementary file 7 — Supplementary Movie 4 [file 41467_2024_45323_MOESM7_ESM.gif]

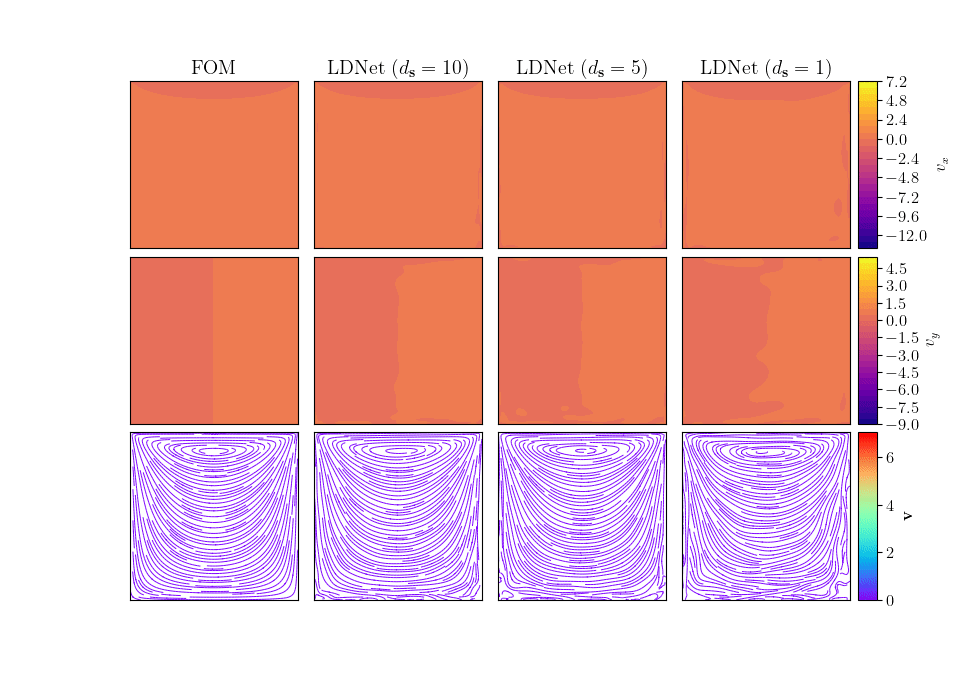

Supplement: Supplementary file 8 — Supplementary Movie 5 [file 41467_2024_45323_MOESM8_ESM.gif]

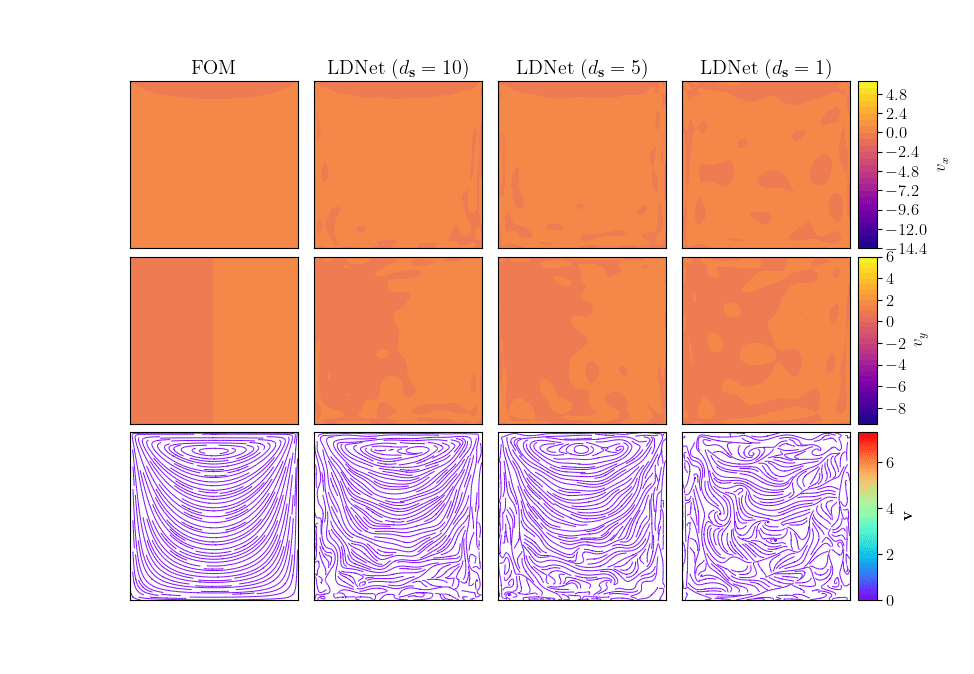

Supplement: Supplementary file 9 — Supplementary Movie 6 [file 41467_2024_45323_MOESM9_ESM.gif]

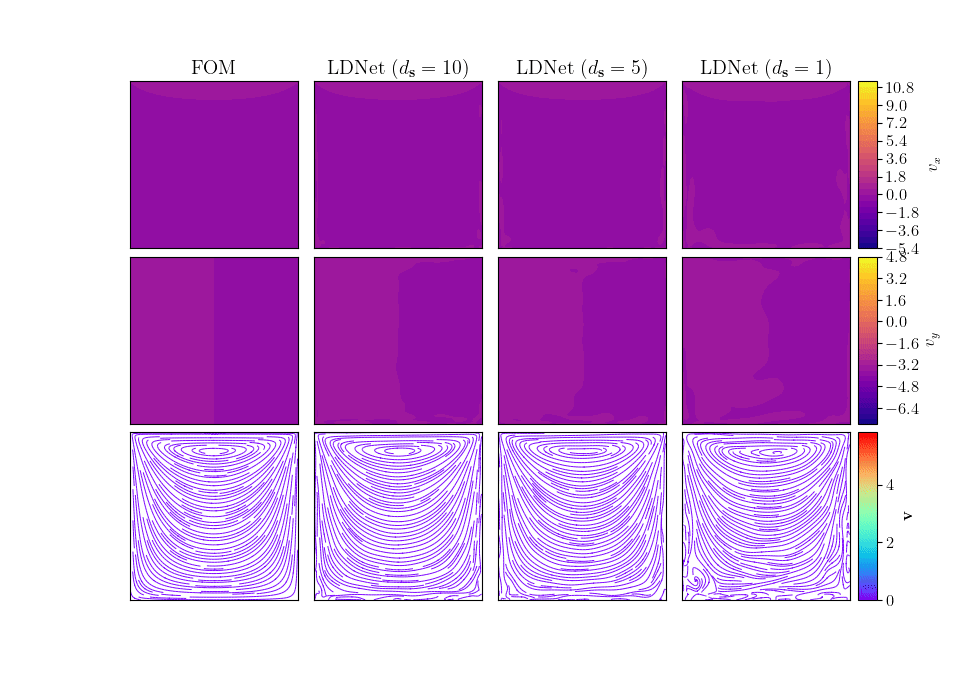

Supplement: Supplementary file 10 — Supplementary Movie 7 [file 41467_2024_45323_MOESM10_ESM.gif]

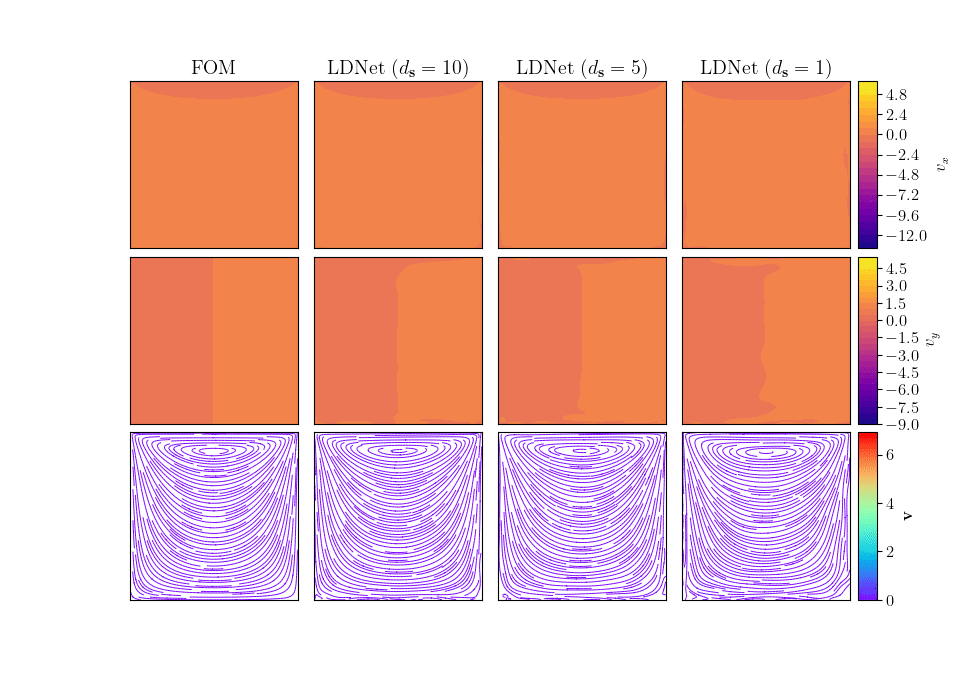

Supplement: Supplementary file 11 — Supplementary Movie 8 [file 41467_2024_45323_MOESM11_ESM.gif]

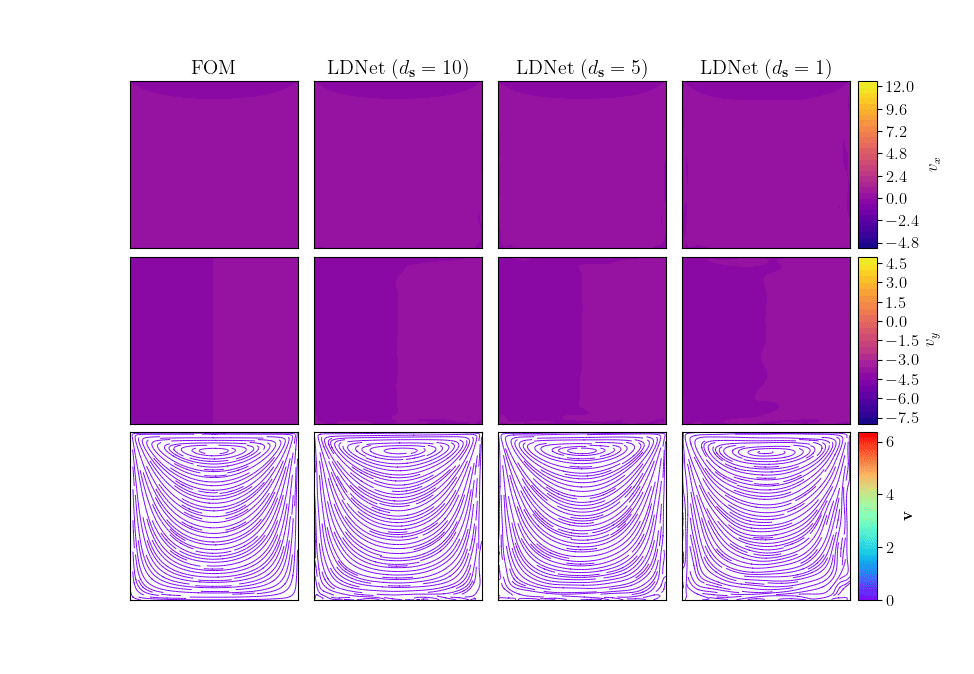

Supplement: Supplementary file 12 — Supplementary Movie 9 [file 41467_2024_45323_MOESM12_ESM.gif]

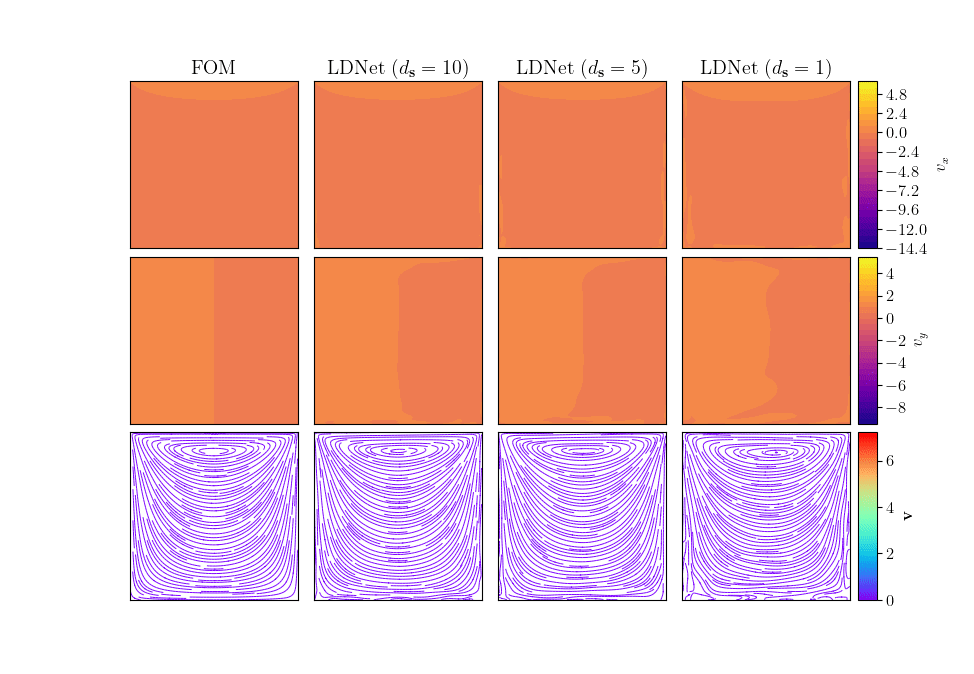

Supplement: Supplementary file 13 — Supplementary Movie 10 [file 41467_2024_45323_MOESM13_ESM.gif]

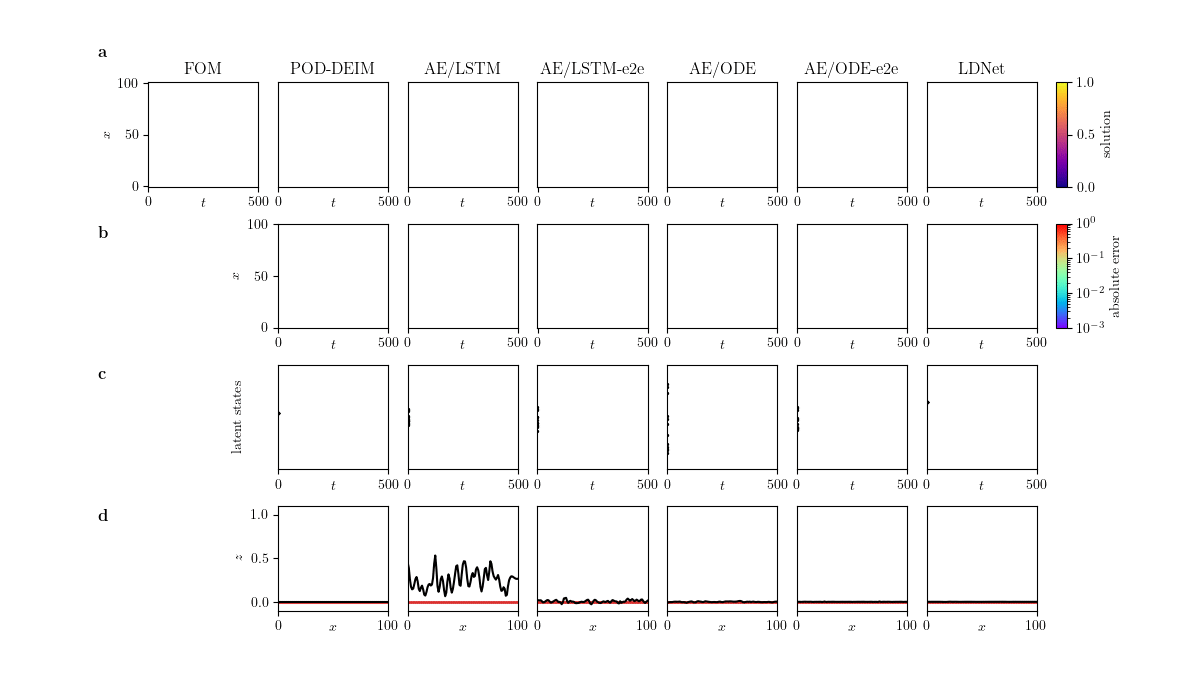

Supplement: Supplementary file 14 — Supplementary Movie 11 [file 41467_2024_45323_MOESM14_ESM.gif]

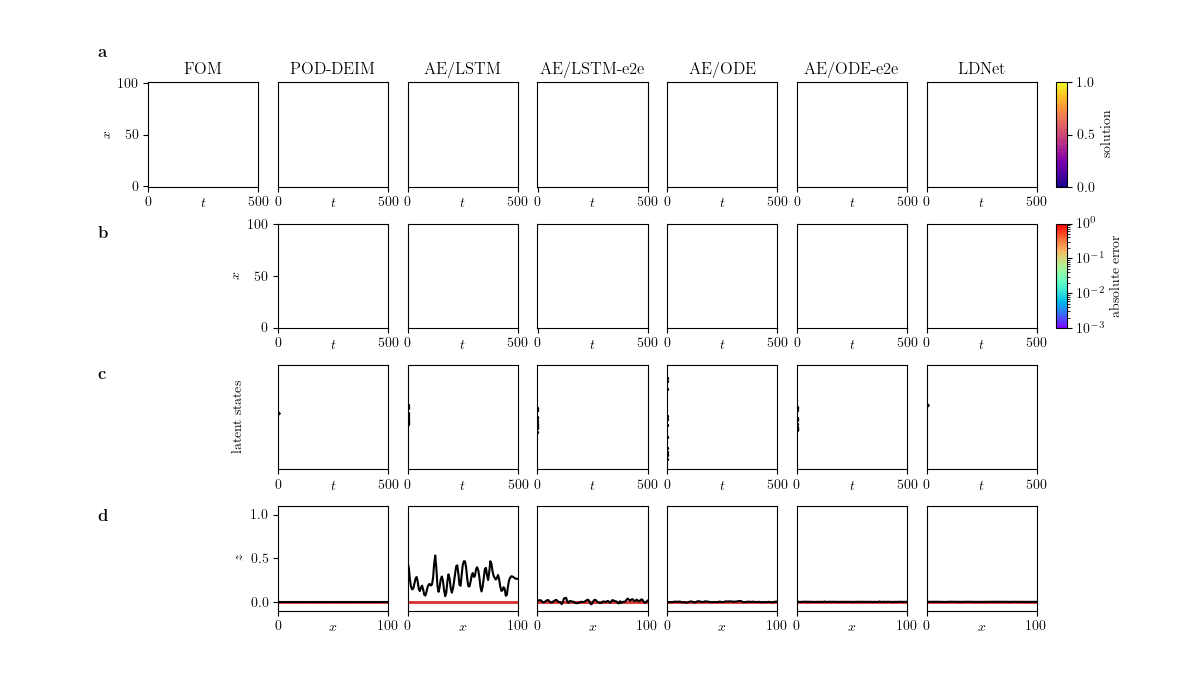

Supplement: Supplementary file 15 — Supplementary Movie 12 [file 41467_2024_45323_MOESM15_ESM.gif]

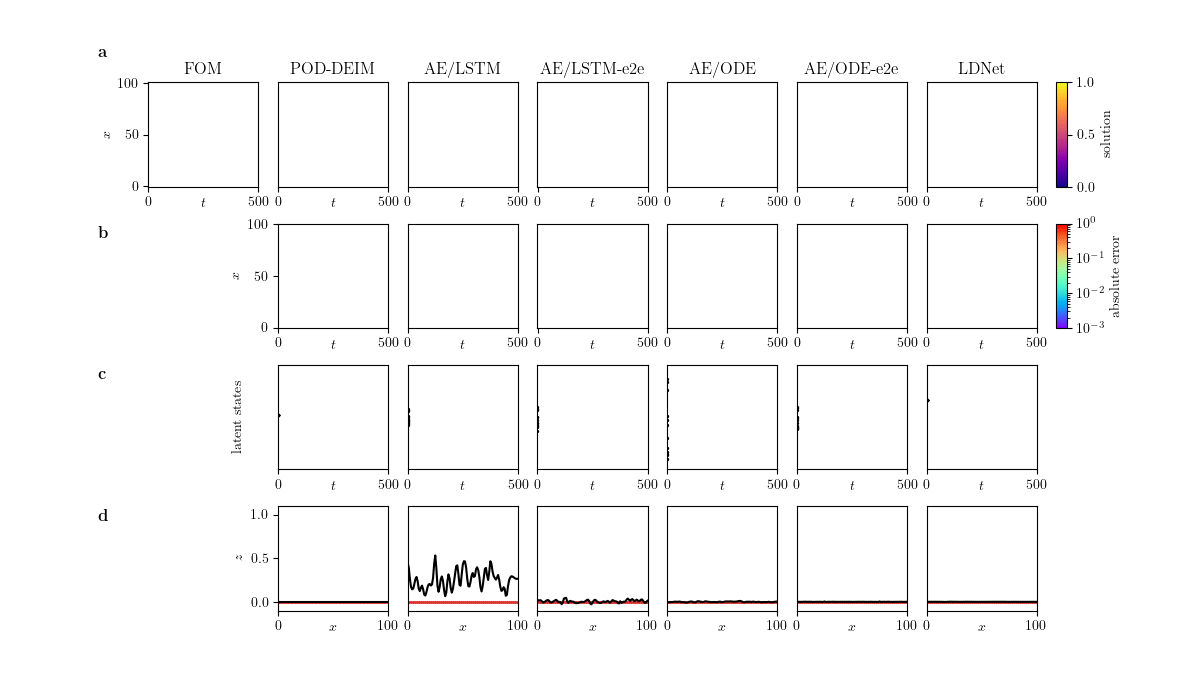

Supplement: Supplementary file 16 — Supplementary Movie 13 [file 41467_2024_45323_MOESM16_ESM.gif]

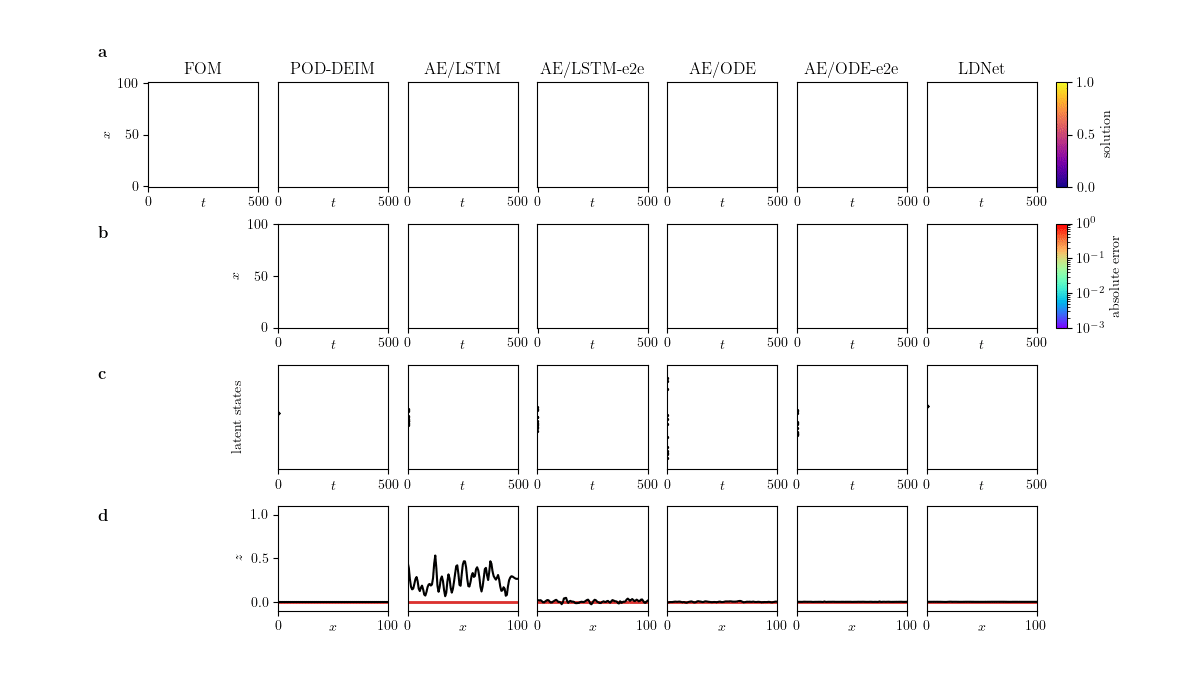

Supplement: Supplementary file 17 — Supplementary Movie 14 [file 41467_2024_45323_MOESM17_ESM.gif]

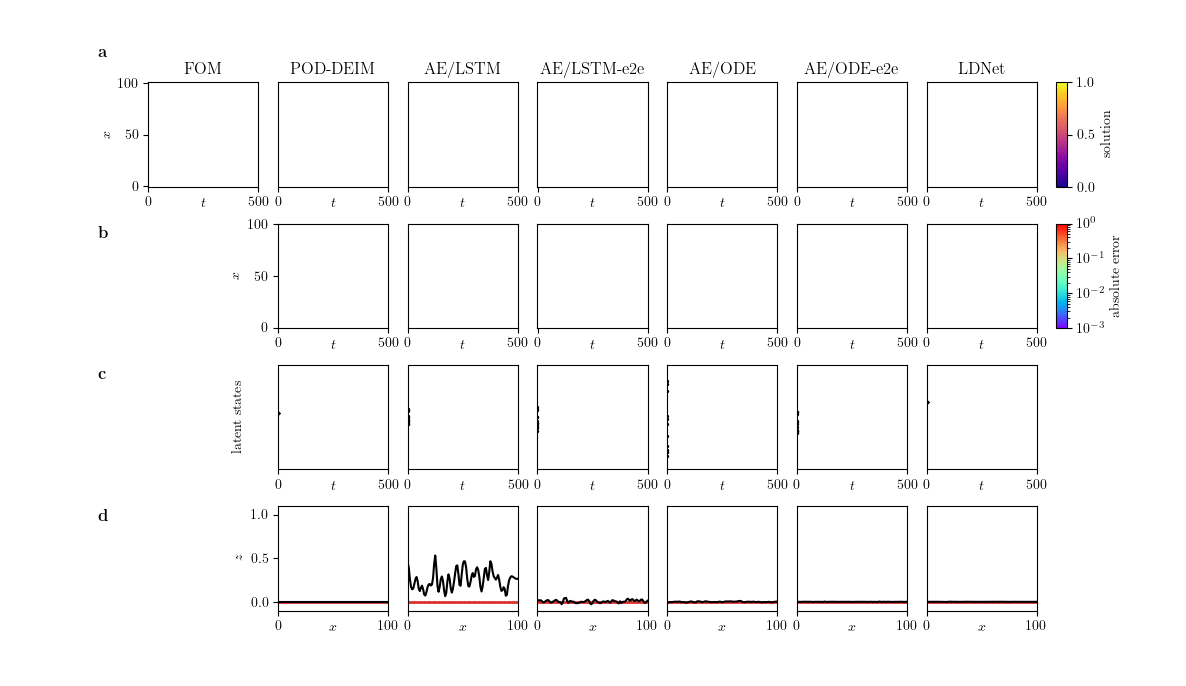

Supplement: Supplementary file 18 — Supplementary Movie 15 [file 41467_2024_45323_MOESM18_ESM.gif]

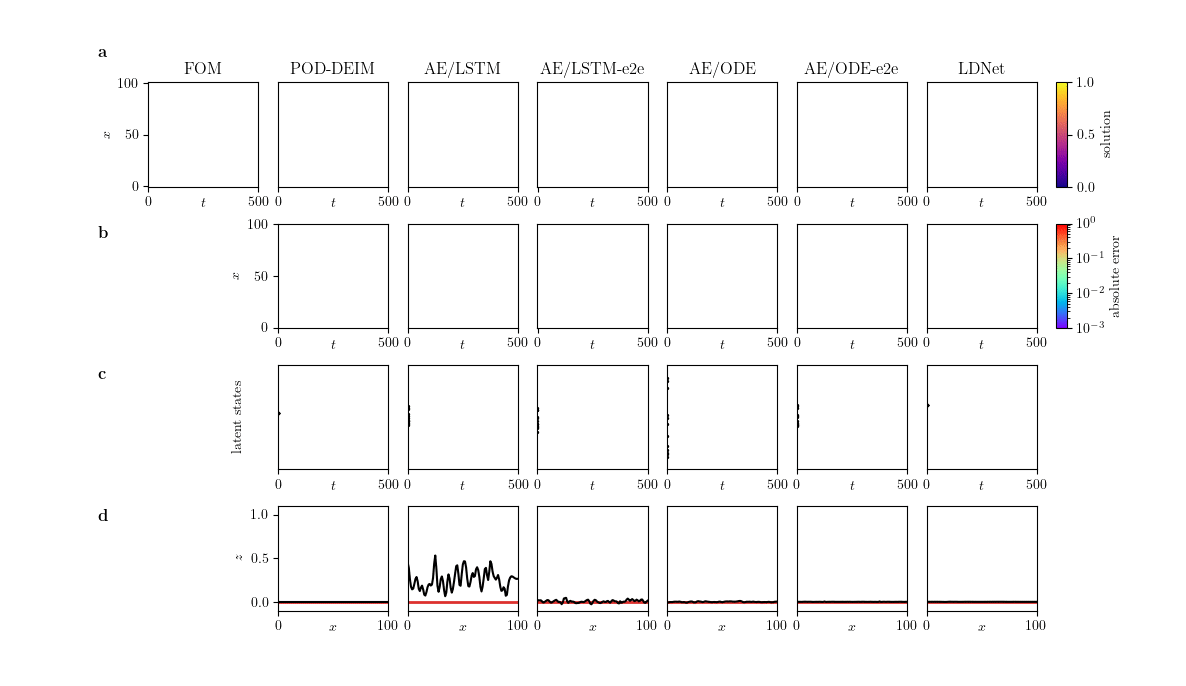

Supplement: Supplementary file 19 — Supplementary Movie 16 [file 41467_2024_45323_MOESM19_ESM.gif]

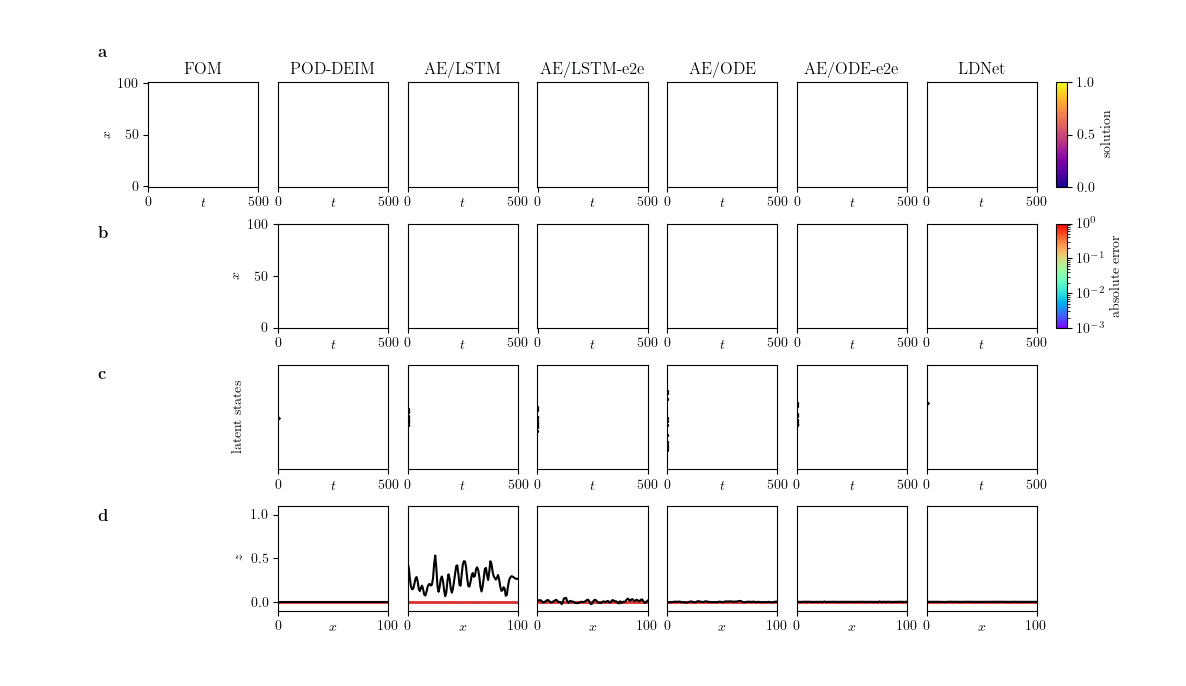

Supplement: Supplementary file 20 — Supplementary Movie 17 [file 41467_2024_45323_MOESM20_ESM.gif]

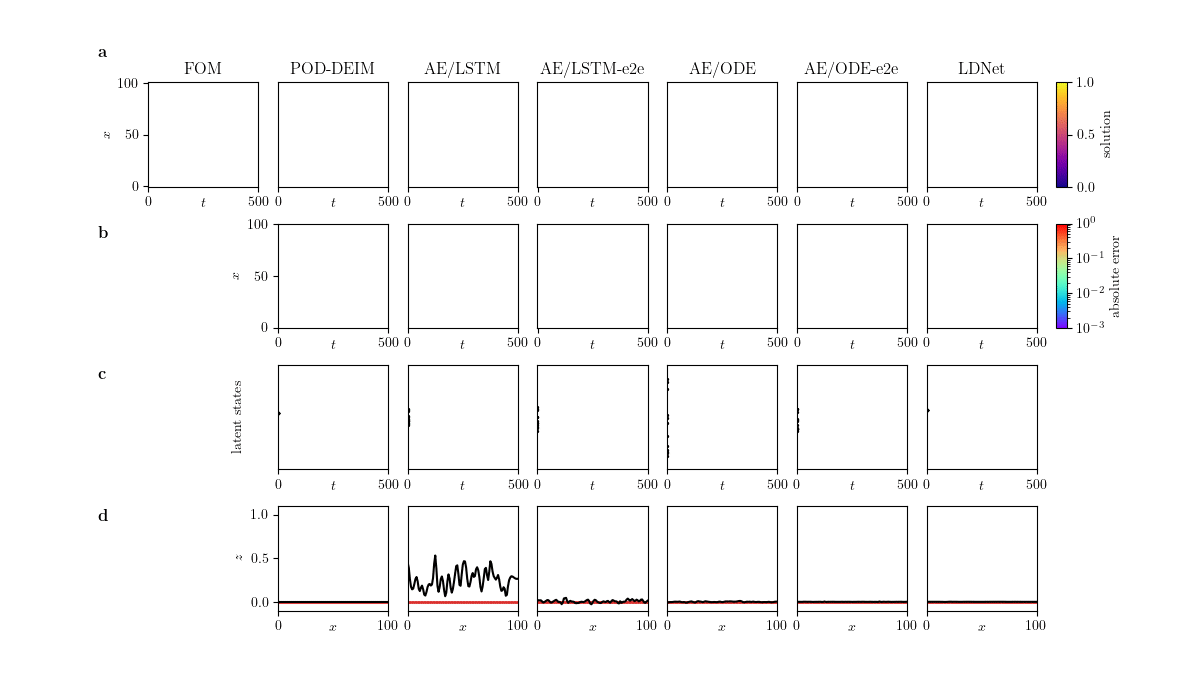

Supplement: Supplementary file 21 — Supplementary Movie 18 [file 41467_2024_45323_MOESM21_ESM.gif]

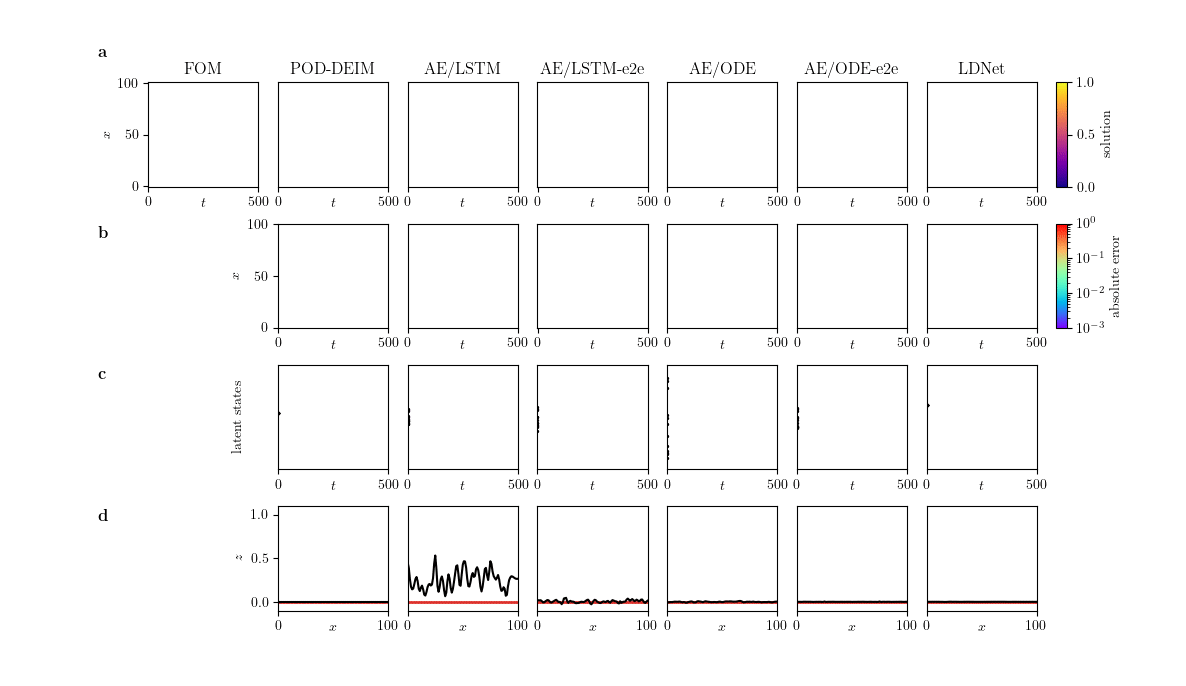

Supplement: Supplementary file 22 — Supplementary Movie 19 [file 41467_2024_45323_MOESM22_ESM.gif]

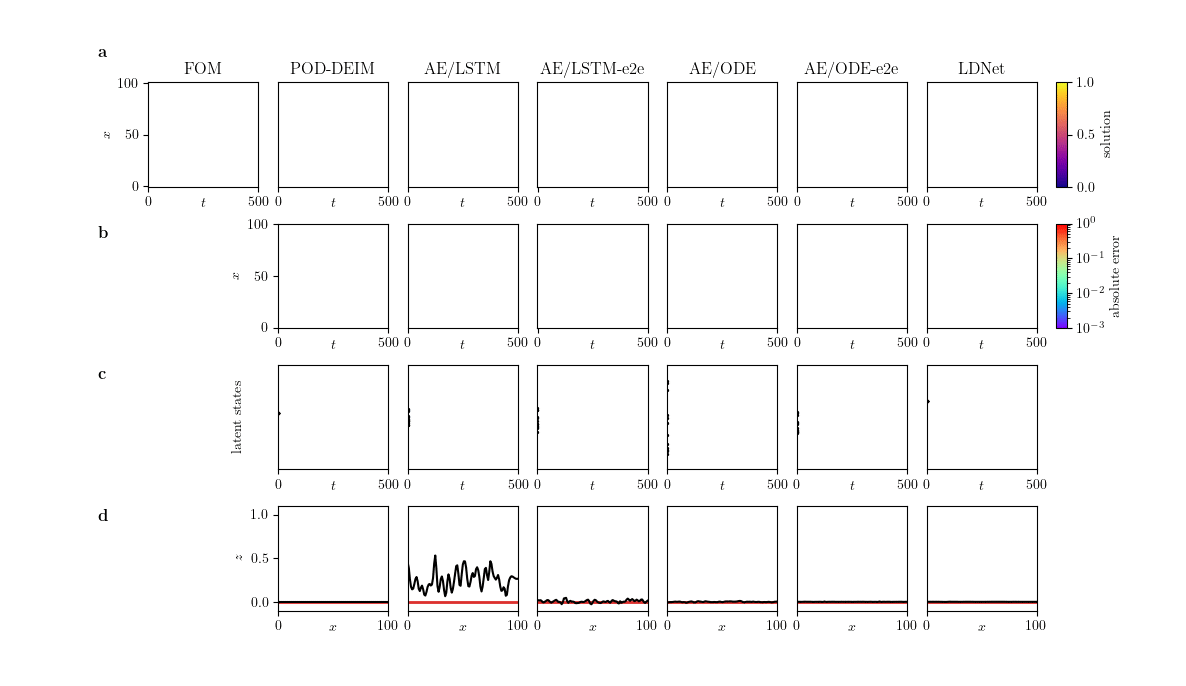

Supplement: Supplementary file 23 — Supplementary Movie 20 [file 41467_2024_45323_MOESM23_ESM.gif]

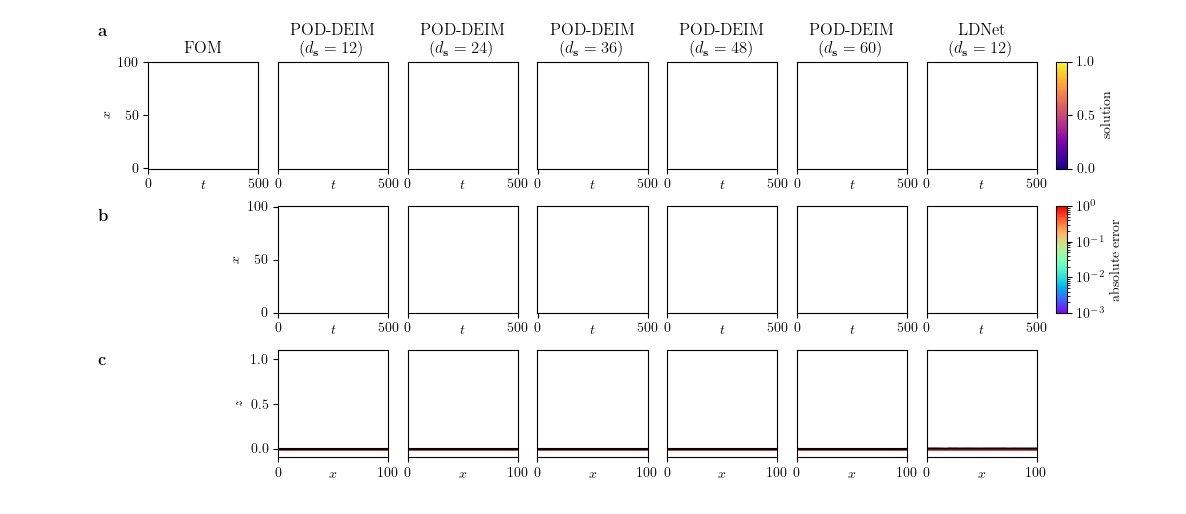

Supplement: Supplementary file 24 — Supplementary Movie 21 [file 41467_2024_45323_MOESM24_ESM.gif]

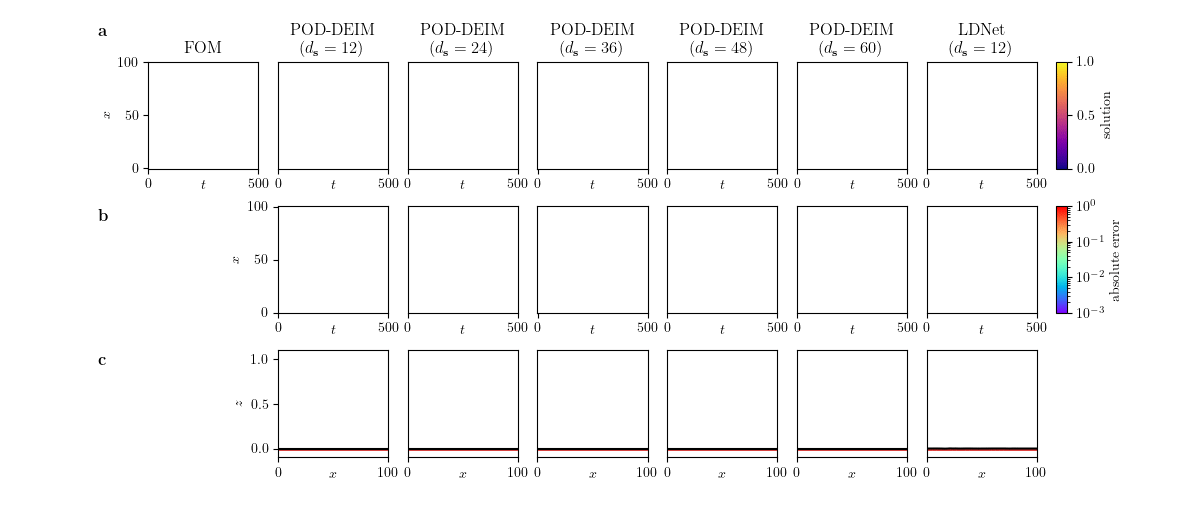

Supplement: Supplementary file 25 — Supplementary Movie 22 [file 41467_2024_45323_MOESM25_ESM.gif]

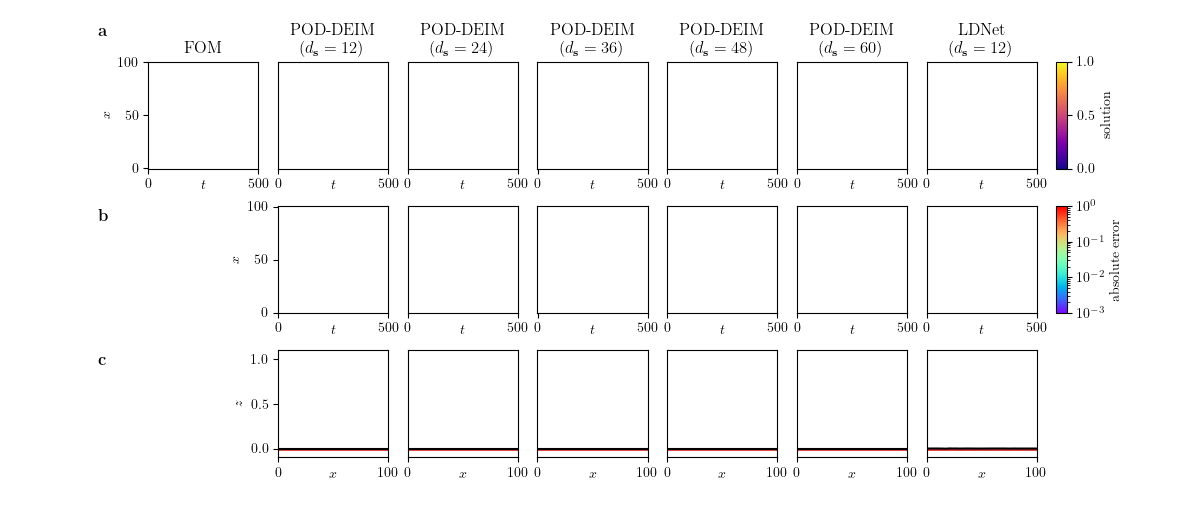

Supplement: Supplementary file 26 — Supplementary Movie 23 [file 41467_2024_45323_MOESM26_ESM.gif]

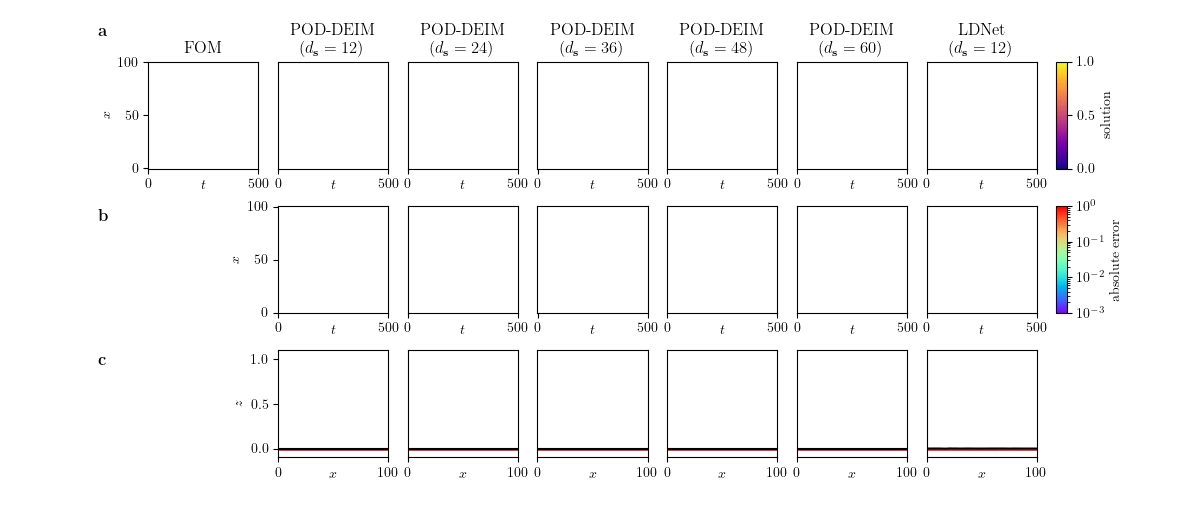

Supplement: Supplementary file 27 — Supplementary Movie 24 [file 41467_2024_45323_MOESM27_ESM.gif]

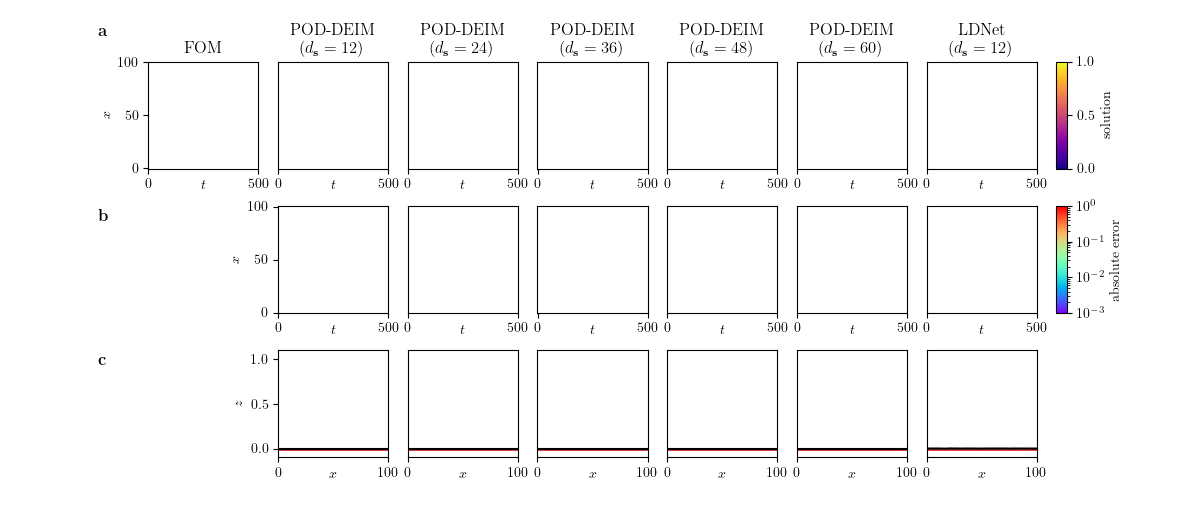

Supplement: Supplementary file 28 — Supplementary Movie 25 [file 41467_2024_45323_MOESM28_ESM.gif]

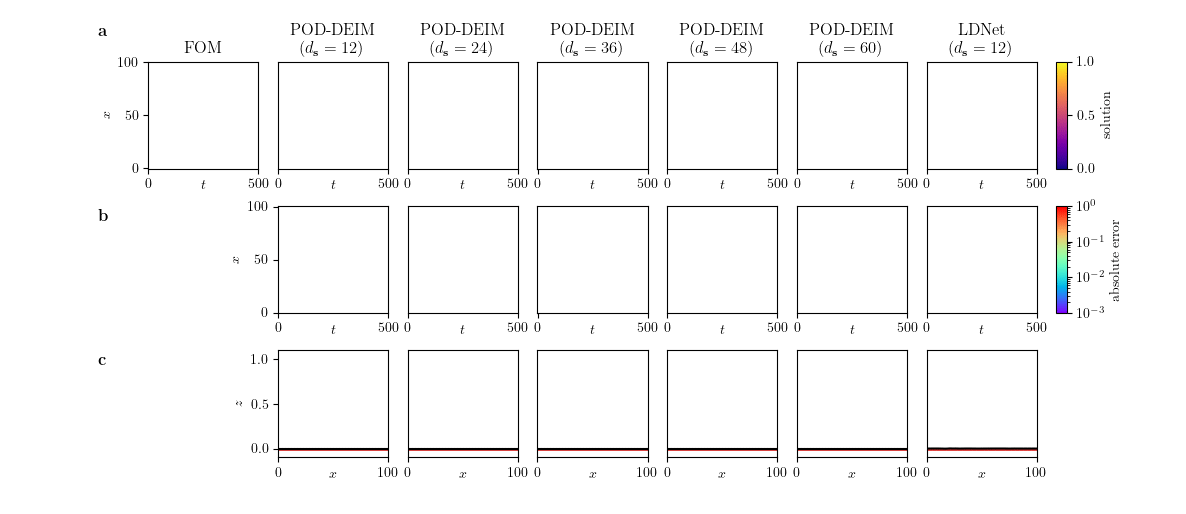

Supplement: Supplementary file 29 — Supplementary Movie 26 [file 41467_2024_45323_MOESM29_ESM.gif]

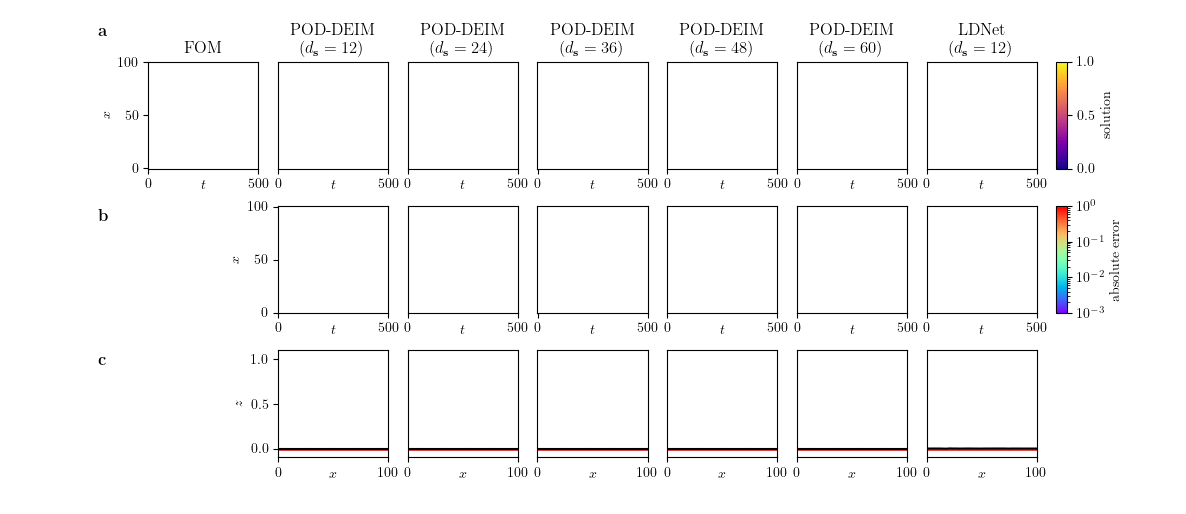

Supplement: Supplementary file 30 — Supplementary Movie 27 [file 41467_2024_45323_MOESM30_ESM.gif]

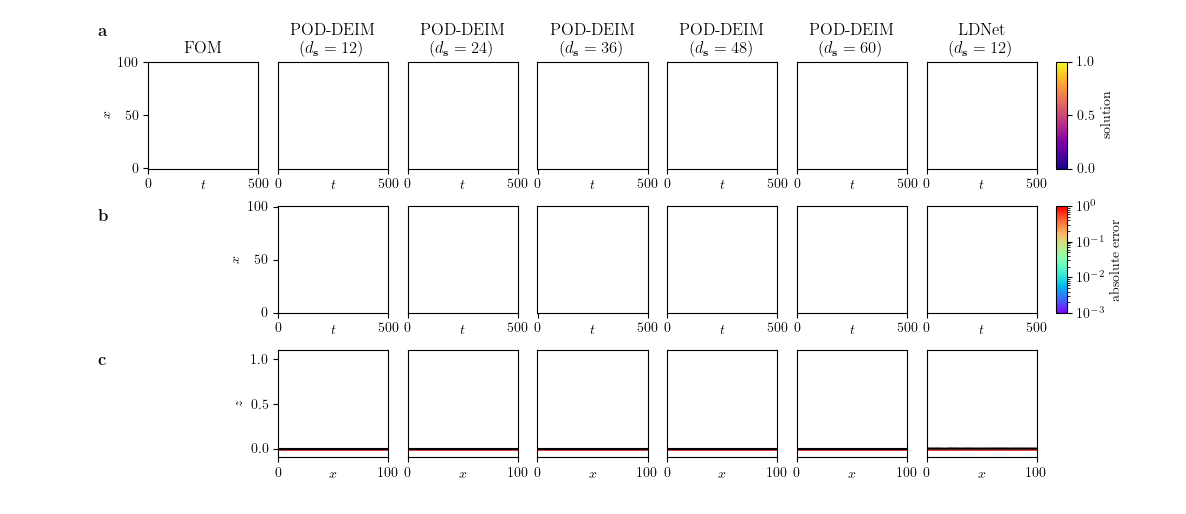

Supplement: Supplementary file 31 — Supplementary Movie 28 [file 41467_2024_45323_MOESM31_ESM.gif]

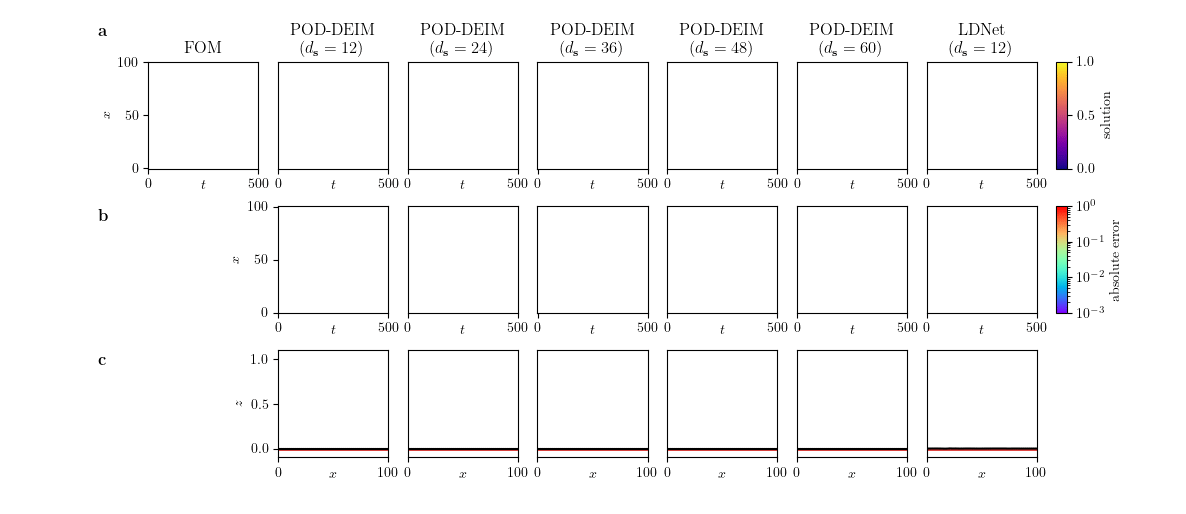

Supplement: Supplementary file 32 — Supplementary Movie 29 [file 41467_2024_45323_MOESM32_ESM.gif]

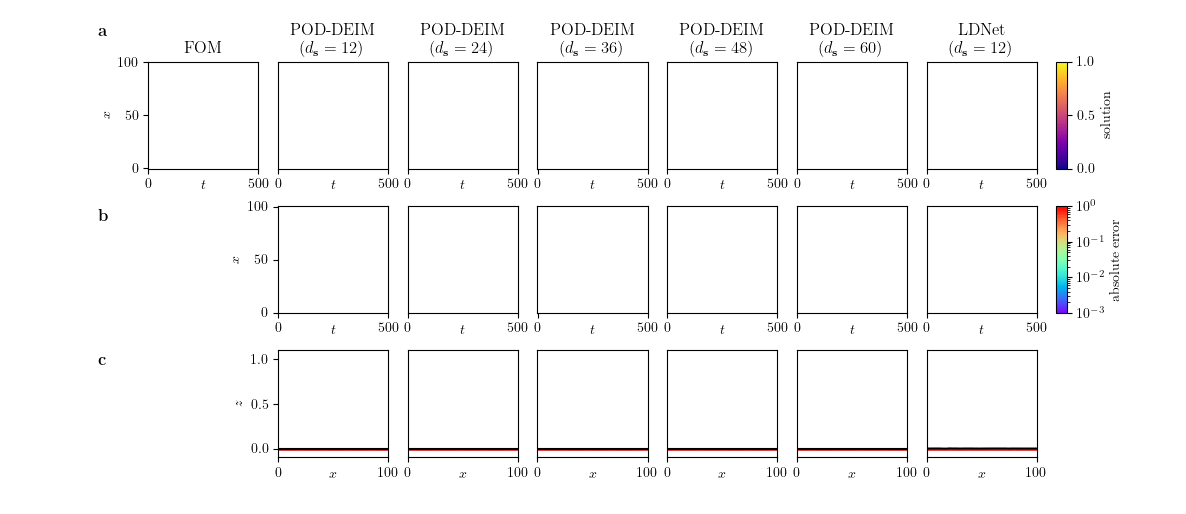

Supplement: Supplementary file 33 — Supplementary Movie 30 [file 41467_2024_45323_MOESM33_ESM.gif]

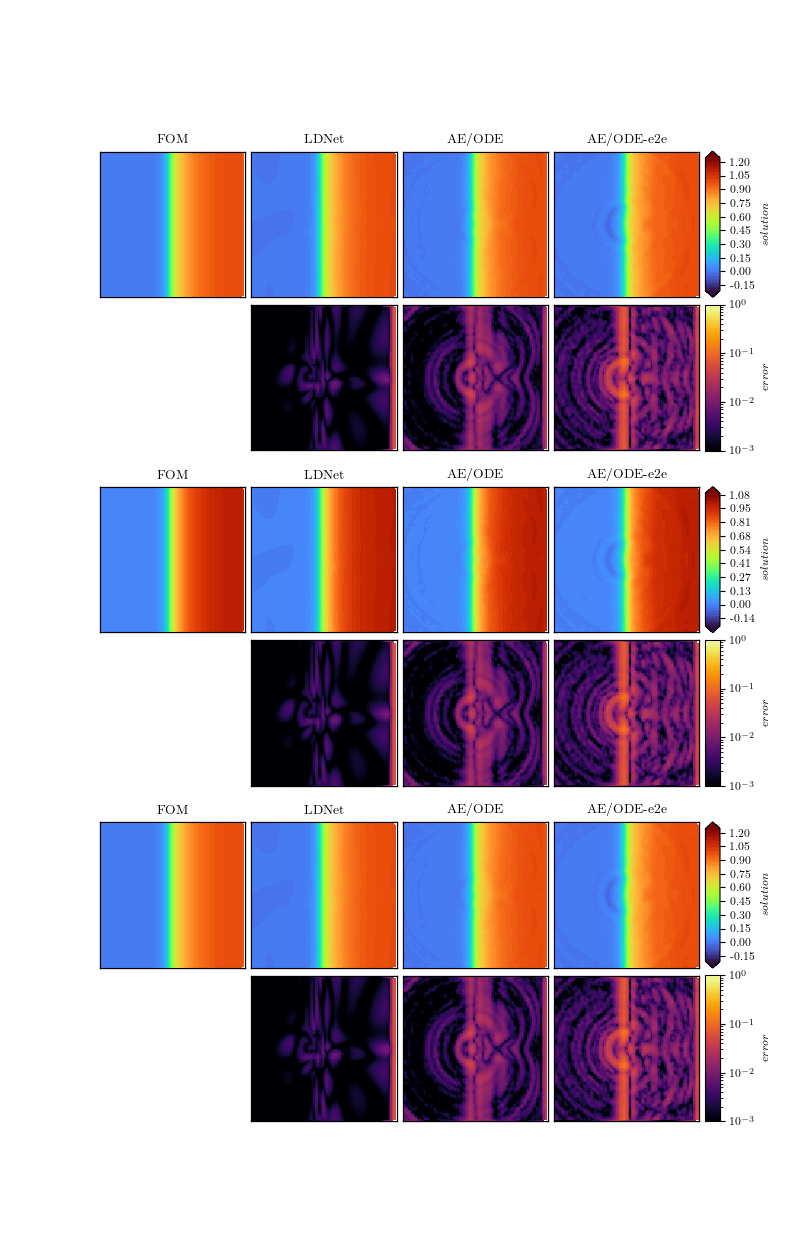

Supplement: Supplementary file 34 — Supplementary Movie 31 [file 41467_2024_45323_MOESM34_ESM.gif]
